# Total Synthesis of 6-Deoxydihydrokalafungin, a Key Biosynthetic Precursor of Actinorhodin, and Its Epimer

**DOI:** 10.3390/molecules26216397

**Published:** 2021-10-22

**Authors:** Takuya Kumamoto, Mika Kainuma, Azusa Takahashi, Yoshika Matsuo, Kazuaki Katakawa, Takaaki Taguchi, Koji Ichinose

**Affiliations:** 1 Department of Synthetic Organic Chemistry, Graduate School of Biomedical and Health Sciences, Hiroshima University, Hiroshima 734-8553, Japan; 2 Research Institute of Pharmaceutical Sciences, Musashino University, Tokyo 202-8585, Japan; knmmka512@gmail.com (M.K.); tkshzss@gmail.com (A.T.); mtsshi66@gmail.com (Y.M.); kazuaki.katakawa@sums.ac.jp (K.K.); ichinose@musashino-u.ac.jp (K.I.); 3 National Institute of Health Sciences, Kanagawa 210-9501, Japan; takatagu@nihs.go.jp

**Keywords:** benzoisochromane, diastereoselective reduction, annulation, deoxydihydrokalafungin, actinorhodin

## Abstract

In this article, we report the total synthesis of 6-deoxydihydrokalafungin (DDHK), a key biosynthetic intermediate of a dimeric benzoisochromanequinone antibiotic, actinorhodin (ACT), and its epimer, *epi*-DDHK. Tricyclic hemiacetal with 3-siloxyethyl group was subjected to Et_3_SiH reduction to establish the 1,3-*cis* stereochemistry in the benzoisochromane, and a subsequent oxidation/deprotection sequence then afforded *epi*-DDHK. A bicyclic acetal was subjected to AlH_3_ reduction to deliver the desired 1,3-*trans* isomer in an approximately 3:1 ratio, which was subjected to a similar sequence to that used for the 1,3-*cis* isomer that successfully afforded DDHK. A semisynthetic approach from (*S*)-DNPA, an isolable biosynthetic precursor of ACT, was also examined to afford DDHK and its epimer, which are identical to the synthetic products.

## 1. Introduction

Actinorhodin (ACT, **1**) is an aromatic polyketide belonging to the dimeric benzoisochromanequinone (BIQ) family [1] and is produced by *Streptomyces coelicolor* A3 (2), which is among the most genetically studied actinomycetes [2]. The biosynthesis of ACT (**1**) includes hydroxylation steps at the C-6 and C-8 positions of the benzoisochromane skeleton [3] mediated via the action of a two-component flavin-dependent monooxygenase (FMO), that is, the ActVA-ORF5/ActVB system comprising the oxygenase ActVA-5 and the flavin: NADH oxidoreductase ActVB. 6-Deoxydihydrokalafungin (DDHK, **3**) was assumed to be the substrate of this FMO, undergoing sequential conversion to the trihydroxynaphthalene and tetrahydroxynaphthalene derivatives T3HN (**4**) and T4HN (**5**), respectively. DDHK (**3**) is presumed to be produced from (*S*)-DNPA (**6**) via reduction by ActVI-2, but has not yet been isolated from any biosynthetic strains of *S. coelicolor* or their mutants because of conversion to the shunt product actinoperylone [4]. To resolve the ambiguity regarding the intermediacy of DDHK (**3**) in ACT biosynthesis, we established a semisynthetic method for obtaining DDHK (**3**) and its epimer *epi*-DDHK (**7**) by the reduction of (*S*)-DNPA (**6**), an isolable biosynthetic precursor of ACT, and successfully clarified the function of the ActVA-ORF5/ActVB system in vitro using semisynthetic DDHK as the substrate [5].

**Scheme 1:**
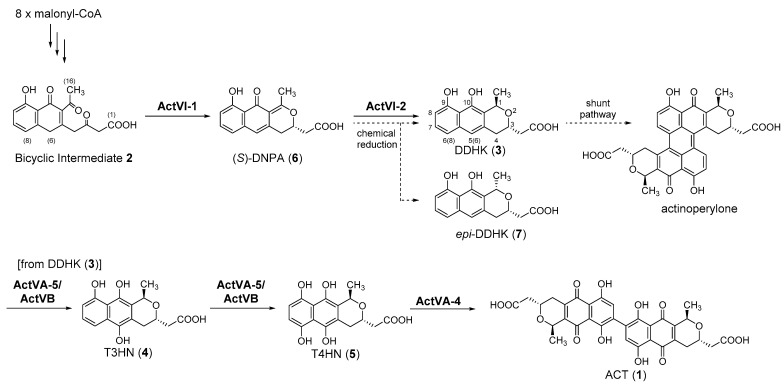
Biosynthetic pathway of ACT (**1**) via (*S*)-DNPA (**6**) and DDHK (**3**). The atom numbering is based on that of the benzoisochromane skeleton. For the numbering in parentheses, see reference [3].

**Scheme 2:**
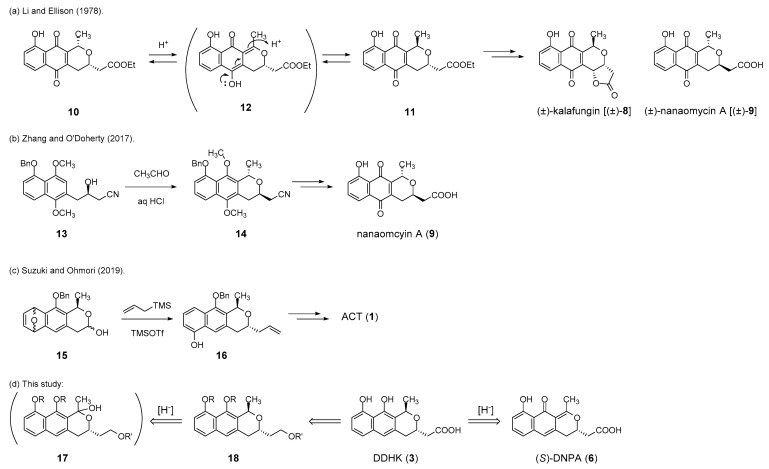
(**a**–**c**) Previous examples of constructing benzoisochromanes with 1,3-*trans* stereochemistry and (**d**) retrosynthesis in this study.

We independently investigated the total synthesis of DDHK (**3**) and its epimer *epi*-DDHK (**7**) for the stereochemical correlation of the semisynthetic products. As shown in Scheme 1, DDHK (**3**) is composed of a benzoisochromane skeleton with two hydroxy groups on the C-9 and C-10 positions [6] and incorporates a disubstituted dihydropyran ring with 1,3-*trans* stereochemistry. In previous reports concerning the synthesis of the biosynthetically related compounds kalafungin (**8**) and nanaomycin A (**9**), which contains a 1,3-*trans*-disubstituted benzoisochromane skeleton as well as a central quinone moiety, epimerization of the 1,3-*cis* isomer to the corresponding *trans* isomer under acidic conditions via conjugation with the C-5 carbonyl group was frequently employed [7,8,9,10,11,12,13,14,15,16,17,18,19,20,21,22,23,24,25]. For example, Li and Ellison [8] reported the total synthesis of racemic kalafungin [(±)-**8**] and nanaomycin A [(±)-**9**], including acid-catalyzed epimerization of 1,3-*cis* **10** to the corresponding *trans* isomer **11** (Scheme 2a). However, this strategy is not suitable for the synthesis of DDHK (**3**) because of the lack of a carbonyl group or other oxygen functionality at the C-5 position. Therefore, a method for direct access to 1,3-*trans*-disubstituted benzoisochromanes is required. Zhang and O′Doherty reported a stereoselective oxa-Pictet–Spengler cyclization of quinol **13** and acetaldehyde for the formation of benzoisochromane **14** with 1,3-*trans* stereochemistry during their synthesis of nanaomycin A (**9**) [26] (Scheme 2b). Furthermore, the group of Suzuki and Ohmori successfully established the 1,3-*trans* stereochemistry in their total synthesis of ACT (**1**) by diastereoselective allylation at the C-3 position of hemiacetal **15** using allylsilane under acidic conditions, in which the stereochemistry was induced by the C-1 stereocenter [27,28] (Scheme 2c). In this article, we report the total synthesis of DDHK (**3**) and its epimer **7** using various reduction conditions for the stereoselective construction of the benzoisochromane moiety. First, we examined the total synthesis of *epi*-DDHK (**7**) with 1,3-*cis* stereochemistry via conventional reduction of cyclic hemiacetal **17**, and we then explored reduction conditions for the construction of 1,3-*trans*-substituted benzoisochromane **18** to achieve the total synthesis of DDHK (**3**). Reduction of (*S*)-DNPA (**6**) to DDHK (**3**) and its epimer **7** was also examined to enable comparison of the spectral data of the synthetic and semisynthetic target compounds (Scheme 2d).

## 2. Results and Discussion

### 2.1. Total Synthesis of epi-DDHK (7)

**Scheme 3:**
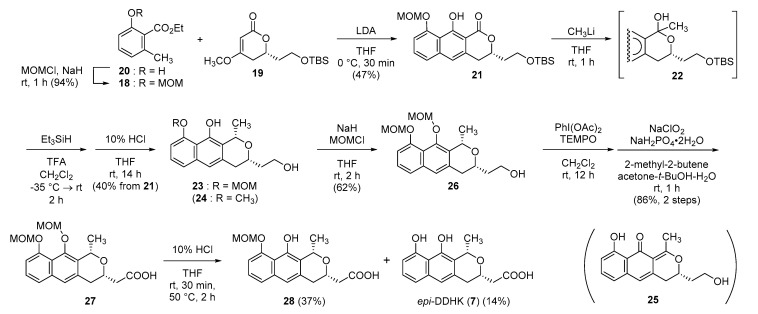
Construction of tricyclic lactone **21** and synthesis of *epi*-DDHK (**7**).

Construction of the key benzoisochromane skeleton of DDHK (**3**) and its epimer **7** was accomplished via Staunton-Weinreb annulation [29,30] using toluate **18** and α,β-unsaturated lactone **19** derived from l-aspartic acid [31], as reported by Donner [22,24] (Scheme 3). The MOM group was selected as the protecting group for phenol **20** to allow for more facile final-stage deprotection under mild acidic conditions compared with methyl group protection. Methylation of lactone **21** with excess CH_3_Li afforded cyclic hemiacetal **22** as a mixture of diastereomers. Treatment of **22** with Et_3_SiH in the presence of TFA in CH_2_Cl_2_ [14,15] followed by deprotection of the TBS group with HCl delivered the corresponding 1,3-*cis*-substituted benzoisochromane **23** in 40% yield from lactone **21**. The stereochemistry of **23** was confirmed by the similarity of its NMR data (see Supplementary Material) to the corresponding methoxy derivative **24** [24]. In this reaction, cyclic enone **25** bearing a hydroxyethyl side chain was obtained as a byproduct, the formation of which was ascribed to dehydration of unreacted hemiacetal **22**. After MOM protection of the phenolic OH in **23**, stepwise oxidation of alcohol **26** using TEMPO followed by Pinnick oxidation [24] afforded carboxylic acid **27**. Finally, removal of the MOM groups under acidic conditions furnished the desired *epi*-DDHK (**7**, 14%) alongside mono-MOM-protected *epi*-DDHK (**28**, 37%).

### 2.2. Stereoselective Reduction Trials Using Modified Silane Reduction Conditions

**Scheme 4:**
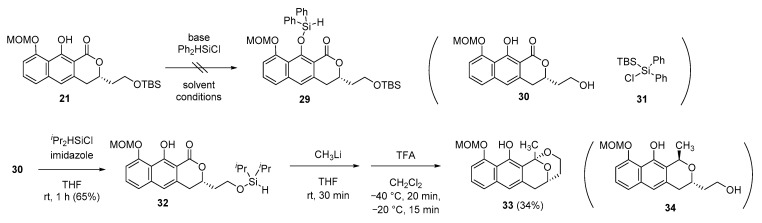
Trials for alternative silane reduction.

Next, the diastereoselective reduction of hemiacetal **22** was examined using several silane reagents for the formation of *trans*-substituted benzoisochromanes. The attempted utilization of Ph_3_SiH instead of Et_3_SiH for the reduction of **22** afforded no reduced products, which was presumably attributable to steric hindrance (data not shown). Similarly, efforts to introduce the silane group into the phenolic OH moiety of **21** using diphenylchlorosilane to prepare silane **29** in the presence of bases such as imidazole and Hünig’s base resulted in no reaction (Scheme 4). When NaH was applied as the base, desilylated alcohol **30** and disilane **31** [32], the formation of which was ascribed to reaction between the TBS group of **21** and the diphenylchlorosilyl anion, were obtained. Desilylated alcohol **30** was further modified by the attachment of a diisopropylsilyl group, and the resulting lactone **32** was methylated and then treated with TFA in order to promote intramolecular delivery of hydride from the silane moiety to the α face of the presumed oxonium ion derived from lactol. However, only bicyclic acetal **33** was obtained, rather than the desired reduced product **34**.

### 2.3. Stereoselective Reduction Trials with Bicyclic Acetal and Total Synthesis of DDHK (3)

**Scheme 5:**
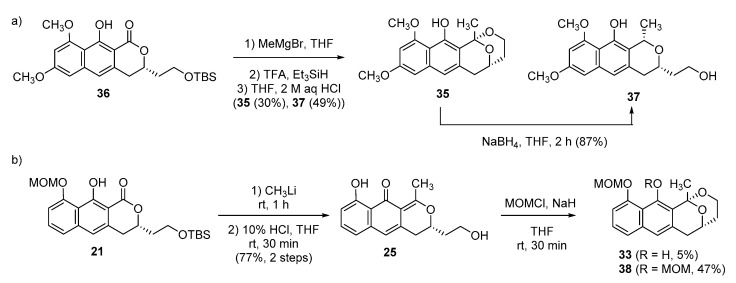
(**a**) Reported generation of bicyclic acetal **35** and NaBH_4_ reduction by Donner et al. [24] and (**b**) conversion of lactone **21** to bicyclic acetals **33** and **38**.

For the construction of the benzoisochromane skeleton bearing 1,3-*trans* stereochemistry, we focused on the aforementioned bicyclic acetal **33** (Scheme 4). The similar bicyclic acetal **35** was reported by Donner as a byproduct of the Et_3_SiH reduction of the hemiacetal derived from lactone **36** to 1,3-*cis* benzoisochromane **37** during the total synthesis of 5-*epi*-9-methoxykalafungin, where NaBH_4_ reduction of **35** afforded **37** [24] (Scheme 5a). We examined an alternative method for the preparation of these bicyclic acetals. Methylation of lactone **21** followed by acid treatment delivered cyclic enone **25** in 77% yield over two steps (Scheme 5b). Subsequent treatment of **25** with excess NaH and MOMCl led to the formation of bicyclic acetal **33** (5%) and the corresponding *bis*-MOM ether **38** (47%). Therefore, reduction trials using major component **38** were performed to explore the construction of the desired 1,3-*trans* stereochemistry.

The reduction of **38** under acidic conditions, which would affect the acetal group, was examined. Application of NaBH_3_CN in the presence of aqueous HCl afforded a mixture of diastereomers (*cis*:*trans* = 81:19, as determined by ^1^H-NMR for the crude product, see Supplementary Material), where the 1,3-*cis* isomer **26** and the desired *trans* isomer **39** were isolated in 69% and 13% yield, respectively (Table 1, run 1). The *trans* stereochemistry of **39** was confirmed by NOE experiments, where enhancement of the signals between the C-1 methyl group and C-3 H atom was observed. The *trans* selectivity was slightly improved (*cis*:*trans* = 74:26) when BH_3_·THF was used (run 2). Inspired by the *trans* selectivity reported by Yamamoto and co-workers for the reduction of aliphatic bicyclic acetals using aluminum hydrides [33], we next examined the use of DIBAL-H. The reaction in CH_2_Cl_2_ at −78 °C afforded an approximately 1:1 mixture of diastereomers (run 3), whereas the undesired *cis* isomer was preferentially obtained when Et_2_O was used as solvent (run 4). The reaction in THF exhibited improved selectivity with a *cis*:*trans* ratio of about 2:3 (run 5). As an alternative aluminum hydride reagent, AlH_3_ prepared from LiAlH_4_ and AlCl_3_ was next investigated. Reaction at −78 °C delivered the desired *trans* isomer in an improved *cis*:*trans* ratio of about 1:3, albeit with only low conversion (run 6). Increasing the temperature to −60 °C afforded a similar diastereoselectivity with improved chemical yield (run 7). Extending the reaction time did not further improve the yield (run 8).

**Scheme 6:**
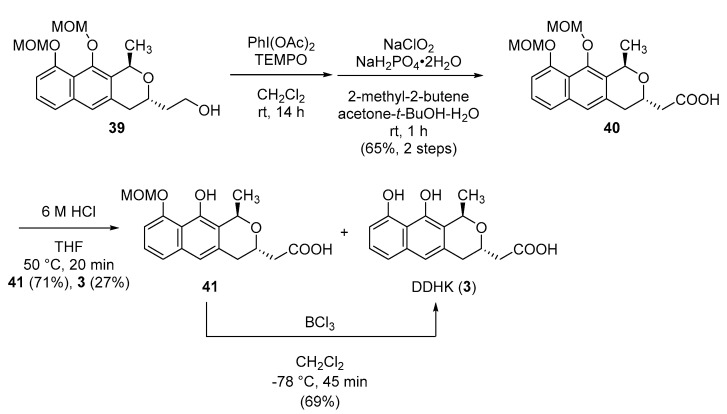
Transformation of alcohol **39** to DDHK (**3**).

The obtained *trans* isomer **39** was subjected to a similar oxidation sequence as *cis* isomer **26** to afford carboxylic acid **40**. Subsequent removal of the MOM groups using 10% HCl in THF for 3 h afforded only a trace amount of DDHK (**3**) alongside the monoprotected DDHK (**41**, 32%). However, adjustment of the acid concentration and reaction time improved the yield of DDHK (**3**) and **41** to 27% and 71%, respectively. Trial of deprotection of mono MOM ether **41** using aqueous HCl resulted in a formation of complex mixture. However, application of BCl_3_ to **41** at −78 °C afforded DDHK (**3**) in 69% yield (Scheme 6).

### 2.4. Semisynthesis of DDHK (3) and Its Epimer 7 from (S)-DNPA (6)

**Scheme 7:**
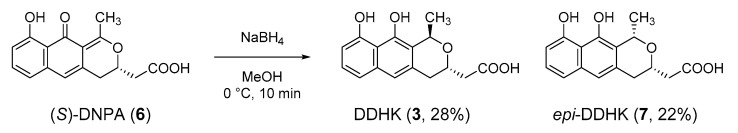
Semisynthesis of DDHK (**3**) and *epi*-DDHK (**7**) from (*S*)-DNPA (**6**).

**Figure 1:**
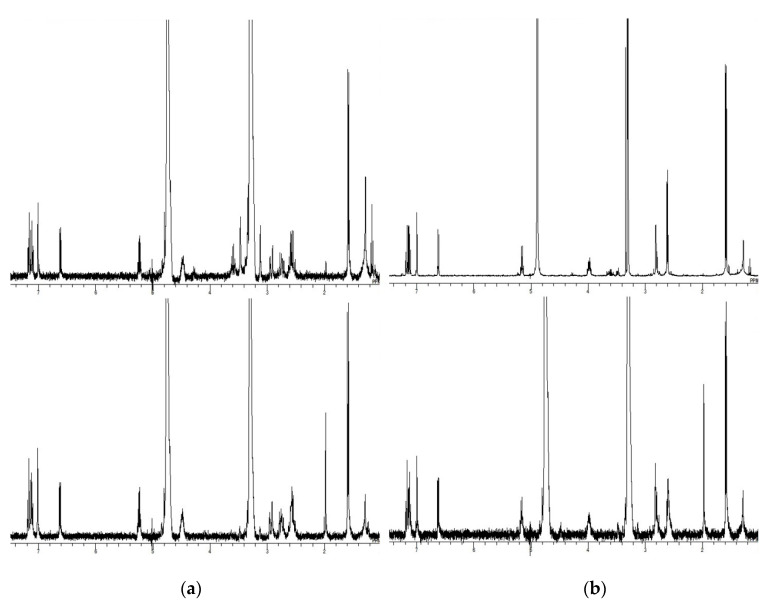
^1^H-NMR spectra (CD_3_OD, 400 MHz) of synthetic and semisynthetic (**a**) DDHK (**3**) and (**b**) *epi*-DDHK (**7**). Top: synthetic; bottom: semisynthetic.

To confirm the structures of the synthetic DDHK (**3**) and its epimer **7**, (*S*)-DNPA (**6**) was isolated from a transformant of *S. coelicolor* [34] and then subjected to NaBH_4_ reduction in methanol (Scheme 7). The crude product consisting of an approximately 1:1 mixture of diastereoisomers was purified by reverse-phase HPLC to afford DDHK (**3**) and its epimer **7**. The semisynthetic products were identified by comparison of their spectral data with those of synthetic **3** and **7** (Figure 1). DDHK (**3**) was observed to be more polar than *epi*-DDHK (**7**).

## 3. Materials and Methods

### 3.1. General

Commercially available reagents and anhydrous solvents were used without further purification. Anhydrous solvents (CH_2_Cl_2_, DMF, and THF) were purchased from FUJIFILM Pure Wako Chemicals (Osaka, Japan) and Kanto Chemical (Tokyo, Japan) Analytical thin-layer chromatography was performed on silica gel 60 F254 plate from Merck (Darmstadt, Germany). Flash chromatography was carried out with Silica gel 60 (100–210 μm) or 60N (neutral, 40–50 μm) from Kanto Chemical (Tokyo, Japan) or Smart Flash AI-580S from Yamazen (Osaka, Japan).

IR spectra were recorded on JASCO FT/IR-4100 and FT/IR-4600 spectrophotometers (Tokyo, Japan) with NaCl plate or Attenuated Total Reflectance Unit ATR PRO450-S. EI-MS was recorded on a JEOL GC-Mate II (Tokyo, Japan). ESIMS was recorded on a JEOL JMS-T100LP in positive ion mode and Thermo Fisher Scientific LTQ Orbitrap XL (Waltham, MA, USA) in positive mode. Optical rotation was measured by JASCO P-1020 and DIP-1000 polarimeters (Tokyo, Japan) ^1^H and ^13^C-NMR spectra were recorded on JEOL ECX 400 (400 MHz for ^1^H-NMR and 100 MHz for ^13^C-NMR) and a JEOL LA 500 spectrometers (125 MHz for ^13^C-NMR)). Chemical shifts were reported in ppm and *J* in Hz. Abbreviations were used for multipilicity: s = singlet, d = doublet, dd = doublets of doublet, ddd = doublets of doublets of doublet, dddd = doublets of doublets of doublets of doublet, t = triplet, quint = quintet, m = multiplet.

### 3.2. Experimental Procedures and Compound Data

*Ethyl 2-(methoxymethoxy)-6-methylbenzoate (**18**).* To a mixture of NaH (55%, 487 mg, 11.2 mmol) in THF (3.0 mL), a solution of **20** (1.00 g, 5.55 mmol) in THF (7.0 mL) was added at 0 °C. The mixture was stirred at 0 °C for 30 min. MOMCl (0.55 mL, 7.24 mmol) was added at 0 °C, and the whole was stirred at rt for 1 h. Saturated aqueous NaHCO_3_ (10 mL) was added, and the whole was partitioned with AcOEt (10 mL) and H_2_O (3 mL). The aqueous layer was extracted with AcOEt (3 mL × 10 mL). Combined organic phase was washed with brine (1 mL × 10 mL) and was dried over Na_2_SO_4_. Solvent was evaporated in vacuo and the residue was purified by column chromatography (CC) (hexane–AcOEt = 95:5–82:18) to give **18** as a colorless oil (1.17 g, 94%). IR (ATR) ν_max_ cm^−1^: 1725 (C=O). ^1^H-NMR (400 MHz, CDCl_3_) δ: 1.38 (3H, t, *J* = 7.3 Hz, CO_2_CH_2_C*H*_3_), 2.31 (3H, s, C6-C*H*_3_), 3.45 (3H, s, OC*H*_3_), 4.40 (2H, q, *J* = 7.3 Hz, CO_2_C*H*_2_), 5.18 (2H, s, OC*H*_2_O), 6.85 (1H, d, *J* = 7.8 Hz, C3-*H*), 6.99 (1H, d, *J* = 8.2 Hz, C5-*H*), 7.22 (1H, dd, *J* = 8.2, 7.8 Hz, C4-*H*). ^13^C-NMR (125 MHz, CDCl_3_) δ:14.2, 19.1, 56.0, 61.0, 94.5, 112.1, 123.4, 125.0, 130.0, 136.2, 153.7, 168.1. HRESIMS *m/z* 247.0940 (calcd for C_12_H_16_NaO_4_: 247.0946).

*(S)-3-{2-[(tert-Butyldimethylsilyl)oxy]ethyl}-10-hydroxy-9-(methoxymethoxy)-3,4-dihydro-1H-benzo[g]isochromen-1-one (**21**).* To a solution of diisopropylamine (0.74 mL, 5.28 mmol) in THF (12 mL), BuLi (1.63 M in hexane, 3.25 mL, 5.30 mmol) was added at −78 °C, and the mixture was stirred at −78 °C for 20 min. A solution of **18** (398 mg, 1.77 mmol) in THF (1.7 mL) was added at −78 °C, and the whole was stirred at −78 °C for 15 min. A solution of **19** (504 mg, 1.76 mmol) in THF (1.7 mL) was added at −78 °C, and the mixture was stirred at 0 °C for 30 min. Saturated aqueous NH_4_Cl (15 mL) was added at 0 °C, and the whole was partitioned with AcOEt (15 mL) and H_2_O (5 mL). Aqueous layer was extracted with AcOEt (2 mL × 15 mL). Combined organic layer was washed with brine (1 mL × 15 mL) and was dried over Na_2_SO_4_. The solvent was evaporated in vacuo, and the residue was purified by CC (hexane–AcOEt = 91:9) to give **21** as a yellow oil (360 mg, 47%). IR (ATR) ν_max_ cm^−1^: 3200–2800 (OH), 1655 (C=O). ^1^H-NMR (400 MHz, CDCl_3_) δ: 0.08 (6H, s, Si(C*H*_3_)_2_), 0.89 (9H, s, SiC(C*H*_3_)_3_), 1.95 (1H, dddd, *J* = 14.2, 8.2, 5.0, 5.0 Hz, one of C1′-*H*_2_), 2.10 (1H, dddd, *J* = 14.2, 8.2, 5.0, 5.0 Hz, one of C1′-*H*_2_), 3.01–3.12 (2H, m, C4-*H*_2_), 3.61 (3H, s, OC*H*_3_), 3.82 (1H, ddd, *J* = 10.5, 10.5, 5.3 Hz, one of C2′-*H*_2_), 3.90 (1H, ddd, 10.5, 8.2, 4.6 Hz, one of C2′-*H*_2_), 4.78 (1H, dddd, *J* = 9.2, 8.0, 4.6, 4.6 Hz, C3-H), 5.38 (2H, s, OC*H*_2_O), 7.01 (1H, s, C5-*H*), 7.10 (1H, dd, *J* = 7.8, 0.9 Hz, C8-*H*), 7.33 (1H, dd, *J* = 7.8, 0.9 Hz, C6-*H*), 7.49 (1H, dd, *J* = 7.8, 7.8 Hz, C7-*H*). ^13^C-NMR (100 MHz, CDCl_3_) δ: −5.48, −5.47, 18.2, 25.8, 33.5, 37.7, 56.4, 58.4, 76.6, 95.8, 102.4, 111.7, 116.0, 116.1, 121.3, 130.5, 133.3, 139.8, 156.3, 163.8, 171.0. [α${]}_{D}^{25}$ + 12.7 (*c* 1.1, CHCl_3_). HREIMS *m/z* 432.1973 (calcd for C_23_H_32_O_6_Si: 432.1968).

*(1S,3S)-2-(3,4-Dihydro-10-hydroxy-9-methoxymethoxy-1-methyl-1H-naphtho[2,3-c]pyran-3-yl)ethanol (**23**) and (S)-3,4-Dihydro-9-hydroxy-3-(2-hydroxyethyl)-1-methyl-10H-naphtho[2,3-c]pyran-10-one (**25**).* To a solution of **21** (102 mg, 0.24 mmol) in THF (1.6 mL), CH_3_Li (1.10 M in Et_2_O, 0.64 mL, 0.70 mmol) was added at 0 °C within 5 min. The whole was stirred at rt for 1 h. Saturated aqueous NH_4_Cl (3 mL) was added at 0 °C, and the whole was partitioned with CH_2_Cl_2_ (6 mL) and H_2_O (2 mL). Aqueous layer was extracted with CH_2_Cl_2_ (3 mL × 6 mL). Combined organic layer was washed with H_2_O (1 mL × 6 mL) and brine (1 mL × 6 mL) and dried over Na_2_SO_4_. Solvent was evaporated in vacuo to give **22** as a yellow oil (110.2 mg, 105%), which was used to next step without further purification. To a solution of crude **22** in CH_2_Cl_2_ (1 mL), TFA (0.054 mL, 0.71 mmol) and Et_3_SiH (0.11 mL, 0.69 mmol) were added successively at −78 °C. The mixture was warmed to −35 °C within 1 h and was stirred at rt for 1 h. Saturated aqueous NaHCO_3_ (5 mL) was added, and the whole was partitioned with CHCl_3_ (10 mL) and H_2_O (3 mL). The aqueous layer was extracted with CHCl_3_ (3 mL × 5 mL). Combined organic layer was washed with H_2_O (1 mL × 5 mL) and brine (1 mL × 5 mL) and was dried over Na_2_SO_4_. Solvent was evaporated in vacuo. The residue (a brown oil, 136 mg) was dissolved in THF (2.5 mL) and 10% HCl (0.5 mL) was added at rt. The mixture was stirred at rt for 14 h. The whole was diluted with H_2_O (3 mL) and was extracted with AcOEt (3 mL × 6 mL). Combined organic layer was washed with brine (1 mL × 6 mL) and was dried over Na_2_SO_4_. Solvent was evaporated in vacuo, and the residue was purified by CC (hexane–AcOEt = 55:45–34:66) to give **23** as a brown oil (30 mg, 40%) and **25** as dark green powder (8 mg, 12%). **23**: IR (ATR) ν_max_ cm^−1^: 3383 (OH). ^1^H-NMR (400 MHz, CDCl_3_) δ: 1.65 (3H, d, *J* = 6.4 Hz, C1-C*H*_3_), 1.88–2.00 (2H, m, C1′-*H*_2_), 2.73 (1H, dd, *J* = 15.6, 1.8 Hz, one of C4-*H*_2_), 2.96 (1H, dd, *J* = 15.6, 11.0 Hz, one of C4-*H*_2_), 3.15 (1H, s, C2′-O*H*), 3.59 (3H, s, OC*H*_3_), 3.83–3.91 (1H, m, C3-*H*), 3.90 (2H, t, *J* = 5.5 Hz, C2′-*H*), 5.26 (1H, q, *J* = 6.4 Hz, C1-*H*), 5.43 (1H, d, *J* = 9.2 Hz, one of OC*H*_2_O), 5.44 (1H, d, *J* = 9.2Hz, one of OC*H*_2_O), 6.98 (1H, dd, *J* = 7.8, 0.9 Hz, C8-*H*), 7.06 (1H, s, C5-*H*), 7.24 (1H, dd, *J* = 8.2, 7.8 Hz, C7-*H*), 7.36 (1H, d, *J* = 7.8 Hz, C6-*H*), 9.61 (1H, s, C10-O*H*). ^13^C-NMR (100 MHz, CDCl_3_) δ: 21.8, 36.0, 37.5, 56.9, 61.6, 71.3, 74.3, 95.8, 107.1, 113.9, 117.5, 121.1, 122.1, 125.5, 134.96, 135.02, 150.0, 153.6. HRESIMS *m/z* 341.1396 (calcd for C_18_H_22_NaO_5_: 341.1365). [α${]}_{D}^{25}$–133.7 (*c* 0.5, CHCl_3_). **25**: mp 114.0–116.3 °C. IR (ATR) ν_max_ cm^−1^: 3290 (O-H), 1635 (C=O). ^1^H-NMR (400 MHz, CDCl_3_) δ: 1.97 (1H, dddd, *J* = 14.6, 7.5, 5.7, 4.8 Hz, one of C1′-*H*_2_), 2.09 (1H, dddd, *J* = 14.6, 7.8, 5.0, 5.0 Hz, one of C1′-*H*_2_), 2.65 (3H, s, C1-C*H*_3_), 2.74 (1H, ddd, *J* = 16.0, 10.8, 1.6 Hz, one of C4-*H*_2_), 2.85 (1H, ddd, *J* = 16.0, 3.4, 0.9 Hz, one of C4-*H*_2_), 3.86–3.95 (2H, m, C2′-*H*), 4.50–4.57 (1H, m, C3-*H*), 6.26 (1H, s, C5-*H*), 6.74 (1H, dd, *J* = 7.8, 0.9 Hz, C8-*H*), 6.78 (1H, dd, *J* = 8.0, 0.9 Hz, C6-*H*), 7.39 (1H, dd, *J* = 8.0, 8.0 Hz, C7-*H*). ^13^C-NMR (125 MHz, CDCl_3_) δ: 23.4, 33.7, 36.7, 58.7, 111.3, 114.3, 115.7, 116.2, 116.9, 129.1, 135.1, 138.4, 163.5, 177.3, 188.8 (1C: missing, overlapped with a signal of CDCl_3_). HRESIMS *m/z* 273.1140 (calcd for C_16_H_17_O_4_: 273.1127). [α${]}_{D}^{25}$ + 116.8 (*c* 0.3, CHCl_3_).

*(1S,3S)-2-{3,4-Dihydro-3-(2-hydroxyethyl)-9,10-bis(methoxymethoxy)-1-methyl-1H-naphtho[2,3-c]pyran-3-yl}ethanol (**26**).* To a suspension of NaH (55%, 2.2 mg, 0.050 mmol) in THF (0.05 mL), a solution of **23** (14 mg, 0.042 mmol) in THF (0.2 mL) was added at 0 °C, and the whole was stirred at 0 °C for 10 min. A solution of MOMCl (0.0038 mL, 0.050 mmol) in THF (0.2 mL) was added at 0 °C, and the whole was stirred at rt for 2 h. Ice-H_2_O (1 mL) was added, and the whole was extracted with AcOEt (3 mL × 3 mL). Combined organic layer was washed with brine (1 mL × 1 mL) and was dried over Na_2_SO_4_. Solvent was evaporated in vacuo, and the residue was purified by CC (hexane–AcOEt = 57:43) to give **26** as a colorless oil (9 mg, 62%). IR (ATR) ν_max_ cm^−1^: 3419 (O-H). ^1^H-NMR (400 MHz, CDCl_3_) δ: 1.71 (3H, d, *J* = 6.2 Hz, C1-C*H*_3_), 1.90–2.01 (2H, m, C1′-*H*_2_), 2.77 (1H, dd, *J* = 15.6, 2.1 Hz, one of C4-*H*_2_), 2.96 (1H, dd, *J* = 15.6, 11.0 Hz, one of C4-*H*_2_), 2.99 (1H, br, O*H*), 3.57 (3H, s, OC*H*_3_), 3.59 (3H, s, OC*H*_3_), 3.88–3.94 (3H, m, C3-*H*, C2′-*H*_2_), 4.96 (1H, d, *J* = 6.6 Hz, one of C10-OC*H*_2_O), 5.19 (1H, d, *J* = 6.6 Hz, one of C10-OC*H*_2_O), 5.28 (1H, d, *J* = 11.2 Hz, one of C9-OC*H*_2_O), 5.29 (1H, d, *J* = 11.2 Hz, one of C9-OC*H*_2_O), 5.39 (1H, q, *J* = 6.2 Hz, C1-*H*), 7.07 (1H, dd, *J* = 7.8, 0.9 Hz, C8-*H*), 7.30 (1H, dd, *J* = 8.2, 7.8 Hz, C7-*H*), 7.33 (1H, s, C5-*H*), 7.41 (1H, dd, *J* = 8.2, 0.9 Hz, C6-*H*). ^13^C-NMR (100 MHz, CDCl_3_) δ: 22.8, 35.8, 37.5, 56.3, 57.7, 61.5, 72.0, 74.1, 96.1, 101.5, 111.8, 118.9, 122.1, 123.2, 125.9, 130.3, 133.7, 135.9, 149.9, 152.5. HRESIMS *m/z* 363.1817 (calcd for C_20_H_27_O_6_: 363.1808). [α${]}_{D}^{25}$ + 77.0 (*c* 0.5, CHCl_3_).

*(1S,3S)-{9,10-Bis(methoxymethoxy)-3,4-dihydro-1-methyl-1H-naphtho[2,3-c]pyran-3-yl}acetic acid (**27**).* To a solution of **26** (26 mg, 0.073 mmol) in CH_2_Cl_2_ (0.2 mL), PIDA (35 mg, 0.109 mmol) and TEMPO (1.2 mg, 0.0077 mmol) were added and the whole was stirred at rt for 12 h. 5% Aqueous Na_2_S_2_O_3_ (2 mL) was added and the whole was extracted with CHCl_3_ (4 mL × 5 mL). Combined organic layer was washed with brine (1 mL × 5 mL) and was dried over Na_2_SO_4_. Solvent was evaporated in vacuo. The residue (a yellow oil, 61 mg) was dissolved with acetone (0.43 mL), *t*-BuOH (0.86 mL), and H_2_O (0.21 mL). 2-Methyl-2-butene (0.21 mL, 1.98 mmol) and NaH_2_PO_4_·2H_2_O (39 mg, 0.25 mmol) were added. NaClO_2_ (16 mg, 0.18 mmol) was added at rt, and the whole was stirred at rt for 1 h; then, 5% HCl (0.5 mL) was added at rt and the whole was extracted with CHCl_3_ (4 mL × 5 mL). Combined organic layer was washed with brine (1 mL × 5 mL) and dried over Na_2_SO_4_. Solvent was evaporated in vacuo, and the residue was purified by CC (CHCl_3_–MeOH = 99:1–91:9) to give **27** as a yellow oil (24 mg, 86%). IR (ATR) ν_max_ cm^−1^: 3500–2900 (O-H), 1711 (C=O). ^1^H-NMR (400 MHz, CDCl_3_) δ: 1.71 (3H, d, *J* = 6.2 Hz, C1-C*H*_3_), 2.71 (1H, dd, *J* = 15.8, 5.5 Hz, one of C4-*H*_2_), 2.79 (1H, dd, *J* = 15.8, 7.3 Hz, one of C4-*H*_2_), 2.87–2.97 (2H, m, C1′-*H*_2_), 3.57 (3H, s, OC*H*_3_), 3.59 (3H, s, OC*H*_3_), 4.08–4.14 (1H, m, C3-*H*), 4.96 (1H, d, *J* = 6.4 Hz, one of C10-OCH_2_O), 5.20 (1H, d, *J* = 6.4 Hz, one of C10-OCH_2_O), 5.28 (1H, d, *J* = 10.8 Hz, one of C9-OCH_2_O), 5.30 (1H, d, *J* = 10.8 Hz, one of C9-OCH_2_O), 5.42 (1H, q, *J* = 6.4 Hz, C1-*H*), 7.07 (1H, dd, *J* = 7.6, 0.9 Hz, C8-*H*), 7.30 (1H, dd, *J* = 8.2, 7.6 Hz, C7-*H*), 7.34 (1H, s, C5-*H*), 7.41 (1H, d, *J* = 7.6 Hz, C6-*H*). ^13^C-NMR (100 MHz, CDCl_3_) δ: 22.6, 35.3, 40.7, 56.5, 57.7, 69.8, 72.3, 96.1, 101.5, 111.1, 119.0, 122.1, 123.3, 125.9, 130.1, 133.0, 135.9, 149.9, 152.5, 175.3. HRESIMS *m/z* 377.1617 (calcd for C_20_H_24_O_7_: 377.1600). [α${]}_{D}^{25}$ + 77.6 (*c* 1.2, CHCl_3_).

*(1S,3S)-(3,4-Dihydro-10-hydroxy-9-methoxymethoxy-1-methyl-1H-naphtho[2,3-c]pyran-3-yl)acetic acid (**28**) and (1S,3S)-(3,4-dihydro-9,10-dihydroxy-1-methyl-1H-naphtho[2,3-c]pyran-3-yl)acetic acid (**7**).* To a solution of **27** (20 mg, 0.053 mmol) in THF (0.6 mL), 10% HCl (0.1 mL) was added at rt, and the whole was stirred at rt for 30 min and at 50 °C for 2 h. Solvent was evaporated in vacuo, and the residue was partitioned with AcOEt (8 mL) and H_2_O (2 mL). The aqueous layer was extracted with AcOEt (2 mL × 5 mL). Combined organic layer was washed with brine (1 mL × 5 mL) and dried over Na_2_SO_4_. Solvent was evaporated *in vacuo* and the residue was purified by CC (CHCl_3_–MeOH = 91:9–82:18) to give **28** as a yellow oil (6.5 mg, 37%) and **7** as a colorless oil (2.2 mg, 14%). **28**: IR (ATR) ν_max_ cm^−1^: 3384 (O-H), 1711 (C=O). ^1^H-NMR (400 MHz, CDCl_3_) δ: 1.66 (3H, d, *J* = 6.4 Hz, C1-C*H*_3_), 2.72 (1H, dd, *J* = 16.0, 5.0 Hz, one of C4 or C1′-*H*_2_), 2.78 (1H, dd, *J* = 16.0, 7.3 Hz, one of C4 or C1′-*H*_2_), 2.87 (1H, d, *J* = 15.1 Hz, one of C4 or C1′-*H*_2_), 2.92 (1H, dd, *J* = 15.1, 11.0 Hz, one of C4 or C1′-*H*_2_), 3.58 (3H, s, OC*H*_3_), 4.02–4.13 (1H, m, C3-*H*), 5.31 (1H, q, *J* = 6.4 Hz, C1-*H*), 5.42 (1H, d, *J* = 8.7 Hz, one of C9-OC*H*_2_O), 5.44 (1H, d, *J* = 8.7 Hz, one of C9-OC*H*_2_O), 6.98 (1H, d, *J* = 7.3 Hz, C8-*H*), 7.06 (1H, s, C5-*H*), 7.24 (1H, dd, *J* = 8.2, 7.8 Hz, C7-*H*), 7.35 (1H, d, *J* = 8.2 Hz, C6-*H*), 9.63 (1H, s, OH). ^13^C-NMR (100 MHz, CDCl_3_) δ: 21.7, 35.4, 40.6, 56.9, 70.0, 71.8, 95.9, 107.3, 114.0, 117.6, 120.2, 122.1, 125.7, 133.8, 135.0, 150.0, 153.6, 172.6. HRESIMS *m/z* 333.1333 (calcd for C_18_H_21_O_6_: 333.1338). [α${]}_{D}^{25}$–104.1 (*c* 0.2, CHCl_3_). **7**: IR (ATR) ν_max_ cm^−1^: 3206 (O-H), 1704 (C=O). ^1^H-NMR (400 MHz, CD_3_OD) δ: 1.59 (3H, d, *J* = 6.2 Hz, C1-C*H*_3_), 2.61 (2H, d, *J* = 6.4 Hz, C4 or C1′-*H*_2_), 2.79 (1H, dd, *J* = 15.3, 9.4 Hz, one of C4 or C1′-*H*_2_), 2.83 (1H, d, *J* = 15.3 Hz, one of C4 or C1′-*H*_2_), 3.98 (1H, dddd, *J* = 9.4, 6.4, 6.4, 4.1 Hz, C3-*H*), 5.16 (1H, q, *J* = 6.2 Hz, C1-*H*), 6.63 (1H, dd, *J* = 7.3, 1.4 Hz, C8-*H*), 7.00 (1H, s, C5-*H*), 7.13 (1H, dd, *J* = 8.2, 7.3 Hz, C7-*H*), 7.18 (1H, dd, *J* = 8.2, 1.4 Hz, C6-*H*). ^13^C-NMR (100 MHz, CD_3_OD) δ: 22.0, 36.7, 41.9, 71.8, 72.7, 108.2, 118.1, 120.5, 120.7, 126.9, 135.6, 136.8, 144.1, 152.0, 154.6, 175.1. HRESIMS *m/z* 289.1087 (calcd for C_16_H_17_O_5_: 289.1076. [α${]}_{D}^{25}$ − 104.6 (*c* 0.1, CHCl_3_).

*(S)-3,4-Dihydro-3-(2-hydroxyethyl)-10-hydroxy-9-(methoxymethoxy)-1H-naphtho[2,3-c]pyran-1-one (**30**).* To a solution of **21** (84 mg, 0.19 mmol) in DMF (0.5 mL), NaH (55%, 11 mg, 0.25 mmol) was added at 0 °C. After 15 min, a solution of Ph_2_HSiCl (0.041 mL, 0.21 mmol) in DMF (0.5 mL) was added, and the whole was stirred at rt for 1.5 h. Ice-H_2_O (2 mL) was added, and the whole was extracted with AcOEt (3 mL × 15 mL). Combined organic layer was washed with brine (1 mL × 15 mL) and was dried over Na_2_SO_4_. Solvent was evaporated in vacuo, and the residue was purified by CC (hexane–AcOEt = 36:64–15:85) to give **30** as colorless solids (50 mg, 82%) and **31** as a colorless oil (61 mg, quant). **30**: mp 100.3–102.4 °C. IR (ATR) ν_max_ cm^−1^: 3519 (O-H), 1635 (C=O). ^1^H-NMR (400 MHz, CDCl_3_) δ: 1.95 (1H, dddd, *J* = 14.7, 8.2, 5.5, 5.0 Hz, one of C1′-*H*_2_), 2.10 (1H, dddd, *J* = 14.7, 8.2, 5.0, 5.0 Hz, one of C1′-*H*_2_), 3.06 (2H, d, *J* = 7.3 Hz, C4-*H*_2_), 3.60 (3H, s, OCH_3_), 3.89 (1H, ddd, *J* = 11.0, 5.5, 5.0 Hz, one of C2′-*H*_2_), 3.90 (1H, ddd, *J* = 11.0, 8.2, 5.0 Hz, one of C2′-*H*_2_), 4.82 (1H, dtd, *J* = 8.2, 7.3, 4.6 Hz, C3-*H*), 5.37 (2H, s, OC*H*_2_O), 6.99 (1H, s, C5-*H*), 7.09 (1H, dd, *J* = 8.2, 0.9 Hz, C8-*H*), 7.32 (1H, d, *J* = 7.8 Hz, C6-*H*), 7.48 (1H, dd, *J* = 8.2, 7.8 Hz, C7-*H*). ^13^C-NMR (100 MHz, CDCl_3_) δ: 33.5, 37.3, 56.48, 56.51, 58.4, 95.8, 102.3, 111.7, 116.1, 116.2, 121.4, 130.7, 133.1, 139.8, 156.2, 163.9, 170.9. HRESIMS *m/z* 319.1182 (calcd for C_17_H_19_O_6_: 319.1182). [α${]}_{D}^{25}$+16.7 (*c* 1.0, CHCl_3_). **31**: ^1^H-NMR (400 MHz, CDCl_3_) δ: 0.06 (6H, s, Si(C*H*_3_)_2_), 0.88 (9H, s, SiC(C*H*_3_)_3_), 7.32–7.74 (6H, m, Ar-H), 7.59 (4H, dd, *J* = 7.8, 1.8 Hz, Ar-H).

*(3S)-3-(2-{[Bis(2-propyl)silyl]oxy}ethyl)-3,4-dihydro-10-hydroxy-9-(methoxymethoxy)-1H-naphtho[2,3-c]pyran-1-one (**32**).* To a solution of **30** (40 mg, 0.13 mmol) and imidazole (17 mg, 0.25 mmol) in THF (1.6 mL), a solution of chlorodiisopropylsilane (0.027 mL, 0.16 mmol) in THF (0.1 mL) was added, and the whole was stirred at rt for 1 h. H_2_O (2 mL) was added at 0 °C and the whole was extracted with Et_2_O (3 mL × 5 mL). Combined organic layer was washed with H_2_O (1 mL × 5 mL) and brine (1 mL × 5 mL) and was dried over Na_2_SO_4_. Solvent was evaporated in vacuo, and the residue was purified by CC (neutral SiO_2_, hexane–AcOEt = 9:1) to give **32** as a colorless oil (36 mg, 65%). IR (ATR) ν_max_ cm^−1^: 3400–2500 (O-H), 2089 (Si-H), 1655 (C=O). ^1^H-NMR (400 MHz, CDCl_3_) δ: 0.08 (14H, m, Si(*i-Pr*)_2_), 1.98 (1H, dddd, *J* = 13.3, 7.8, 5.5, 5.5 Hz, one of C1′-H_2_), 2.13 (1H, dddd, *J* = 13.3, 7.8, 5.0, 5.0 Hz, one of C1′-H_2_), 3.05 (1H, ddd, *J* = 16.0, 9.2, 0.9 Hz, one of C4-*H*_2_), 3.09 (1H, dd, *J* = 16.0, 4.1 Hz, one of C4-*H*_2_), 3.61 (3H, s, OC*H*_3_), 3.91 (1H, ddd, *J* = 10.1, 5.0, 5.0 Hz, one of C2′-*H*_2_), 3.99 (1H, ddd, *J* = 10.1, 8.2, 5.0 Hz, one of C2′-*H*_2_), 4.81 (1H, dddd, *J* = 9.6, 8.2, 4.6, 4.6 Hz, C3-*H*), 5.37 (2H, s, OCH_2_O), 7.01 (1H, s, C5-*H*), 7.10 (1H, dd, *J* = 7.8, 0.9 Hz, C8-*H*), 7.33 (1H, dd, *J* = 8.2, 0.9 Hz, C6-*H*), 7.49 (1H, dd, *J* = 8.2, 7.8 Hz, C7-*H*), 13.07 (1H, s, O*H*). ^13^C-NMR (100 MHz, CDCl_3_) δ: 12.25, 12.29, 17.3, 17.4, 33.5, 37.7, 56.50, 56.54, 60.9, 95.8, 102.5, 111.7, 116.2 (overlapped), 121.4, 130.7, 133.3, 139.9, 156.3, 163.9, 171.0. HRESIMS *m/z* 433.2067 (calcd for C_23_H_33_O_6_Si: 433.2046). [α${]}_{D}^{25}$ + 14.9 (*c* 1.2, CHCl_3_).

*(1R,5S)-11-Methoxymethoxy-1-methyl-3,4,5,6-tetrahydro-1,5-epoxy-1H-naphtho[2,3-c]oxocin-12-ol (**33**).* To a solution of **32** (33 mg, 0.079 mmol) in THF (0.5 mL), CH_3_Li (1.10 M in Et_2_O, 0.28 mL, 0.31 mmol) was added at 0 °C, and the whole was stirred at rt for 30 min. Saturated aqueous NH_4_Cl (3 mL) was added at 0 °C, and the whole was extracted with CH_2_Cl_2_ (4 mL × 6 mL). The combined organic layer was washed with H_2_O (1 mL × 6 mL) and brine (1 mL × 6 mL) and was dried over Na_2_SO_4_. Solvent was evaporated in vacuo, and the residue (55 mg, 161%) was used for the next step without purification. To a solution of crude hemiacetal (55 mg) in CH_2_Cl_2_ (0.4 mL), TFA (0.02 mL, 0.23 mmol) in CH_2_Cl_2_ (0.1 mL) was added at −78 °C, and the whole was stirred at −40 °C for 20 min and at −20 °C for 15 min. Saturated aqueous NaHCO_3_ (3 mL) was added and the whole was partitioned with CHCl_3_ (6 mL) and H_2_O (1.5 mL). Aqueous layer was extracted with CHCl_3_ (3 mL × 5 mL). Combined organic layer was washed with H_2_O (1 mL × 5 mL) and brine (1 mL × 5 mL) and dried over Na_2_SO_4_. The solvent was evaporated in vacuo, and the residue was purified by CC (hexane–AcOEt = 79:21–59:41) to give **33** as a colorless oil (9 mg, 34%). IR (neat) ν_max_ cm^−1^: 3385 (O-H). ^1^H-NMR (400 MHz, CDCl_3_) δ: 1.40 (1H, dif.dd, *J* = 13.6, 3.2 Hz, one of C6-*H*_2_), 2.07 (3H, s, C1-C*H*_3_), 2.51 (1H, m, one of C6-*H*_2_), 2.79 (1H, d, *J* = 17.4 Hz, one of C4-*H*_2_), 3.56–3.64 (2H, m, one of C4-*H*_2_ and C7-*H*_2_), 3.59 (3H, s, OC*H*_3_), 3.79 (1H, dd, *J* = 11.9, 6.9 Hz, one of C7-*H*_2_), 4.54 (1H, dd, *J* = 6.9, 6.9 Hz, C5-*H*), 5.42 (1H, d, *J* = 15.1 Hz, one of C11-OC*H*_2_O), 5.44 (1H, d, *J* = 15.1 Hz, one of C11-OC*H*_2_O), 7.02 (1H, dd, *J* = 6.9, 0.9 Hz, C10-*H*), 7.11 (1H, s, C7-*H*), 7.28 (1H, t, *J* = 7.8 Hz, C9-*H*), 7.36 (1H, d, *J* = 8.2 Hz, C8-*H*), 10.00 (1H, s, O*H*). ^13^C-NMR (125 MHz, CDCl_3_) δ: 27.1, 29.3, 30.4, 57.1, 59.5, 66.2, 95.9, 96.1, 107.3, 114.4, 117.0, 117.9, 121.9, 126.5, 135.9, 136.1, 151.7, 154.4. HRESIMS *m/z* 317.1384 (calcd for C_18_H_21_O_5_: 317.1389). [α${]}_{D}^{25}$ − 71.7 (c 0.3, CHCl_3_).

*Synthesis of **25**.* To a solution of **21** (274 mg, 0.63 mmol) in THF (3.5 mL), CH_3_Li (1.10 M in Et_2_O, 1.7 mL, 1.87 mmol) was added at 0 °C, and the whole was stirred at rt for 1 h. Saturated aqueous NH_4_Cl (6 mL) was added at 0 °C, and the whole was extracted with CH_2_Cl_2_ (3 mL × 10mL). Combined organic layer was washed with H_2_O (1 mL × 5 mL) and brine (1 mL × 5 mL) and was dried over Na_2_SO_4_. Solvent was evaporated in vacuo to give crude **22** as a yellow oil (285 mg), which was used to next reaction without purification. To a solution of crude **22** in THF (2.5 mL), 10% HCl (0.5 mL) was added at rt, and the whole was stirred at rt for 30 min. H_2_O (9 mL) was added at rt and the whole was extracted with AcOEt (3 mL × 15mL). Combined organic layer was washed with brine (1 mL × 10 mL) and was dried over Na_2_SO_4_. The solvent was evaporated in vacuo, and the residue was washed with a mixed solvent of hexane and AcOEt (1:1) to give **25** as yellow powder (132 mg, 77%). The physical properties of **25** obtained here were identical with those prepared as described above.

*(1R,5S)-11,12-Bis(methoxymethoxy)-1-methyl-3,4,5,6-tetrahydro-1H-1,5-epoxynaphtho[2,3-c]oxocine(****38****).* To a suspension of NaH (60%, 75 mg, 1.87 mmol) in THF (1 mL), **25** (206 mg, 0.76 mmol) in THF (6 mL) was added, and the whole was stirred at 0 °C for 20 min. MOMCl (0.13 mL, 1.71 mmol) was added, and the whole was stirred at rt for 1 h. NaH (60%, 30 mg, 0.75 mmol) and MOMCl (0.06 mL, 0.79 mmol) was added at 0 °C, and the whole was stirred at rt for 30 min. Ice-H_2_O (5 mL) was added, and the whole was extracted with AcOEt (1 mL × 15 mL, 2 mL × 10 mL). Combined organic layer was washed with brine (1 mL × 10 mL) and was dried over Na_2_SO_4_. Solvent was evaporated in vacuo, and the residue was purified by CC (hexane–AcOEt = 76:24–54:45) to give **38** as a yellow oil (128 mg, 47%) and **33** (a yellow oil, 13 mg, 5%). **38**: IR (ATR) ν_max_ cm^−1^: no characteristic absorption. ^1^H-NMR (400 MHz, CDCl_3_) δ: 1.43 (1H, dd, *J* = 13.7, 3.2 Hz, one of C6-*H*_2_), 2.11 (3H, s, C1-C*H*_3_), 2.51 (1H, m, one of C6-*H*_2_), 2.82 (1H, d, *J* = 16.9 Hz, one of C4-*H*_2_), 3.56–3.67 (2H, m, one of C3- and C4-*H*_2_), 3.58 (3H, s, OC*H*_3_), 3.67 (3H, s, OC*H*_3_), 3.75 (1H, dd, *J* = 11.9, 6.9 Hz, one of C3-*H*_2_), 4.55 (1H, dd, *J* = 6.9, 6.9 Hz, C5-*H*), 4.87 (1H, d, *J* = 4.1 Hz, one of C11-OC*H*_2_O), 5.10 (1H, d, *J* = 4.1 Hz, one of C11-OC*H*_2_O), 5.30 (1H, d, *J* = 6.5 Hz, one of C12-OC*H*_2_O), 5.33 (1H, d, *J* = 6.5 Hz, one of C12-OC*H*_2_O), 7.10 (1H, dd, *J* = 7.8, 0.9 Hz, C10-*H*), 7.34 (1H, dd, *J* = 8.2, 7.8 Hz, C9-*H*), 7.37 (1H, s, C7-*H*), 7.40 (1H, dd, *J* = 8.2, 1.3 Hz, C8-*H*). ^13^C-NMR (100 MHz, CDCl_3_) δ: 27.4, 29.3, 33.0, 53.4, 56.5, 58.9, 65.7, 95.9, 96.3, 100.7, 111.6, 120.0, 121.7, 122.5, 126.7, 127.5, 134.7, 136.5, 149.7, 153.4. HRESIMS *m/z* 361.1658 (calcd for C_20_H_25_O_6_: 361.1651). [α${]}_{D}^{25}$ − 216.1 (*c* 1.5, CHCl_3_).

*(1R,3S)-2-[3,4-Dihydro-9,10-bis(methoxymethoxy)-1-methyl-1H-naphtho[2,3-c]pyran-3-yl]ethanol (**39**). (*Table 1*run 7).* To a suspension of LiAlH_4_ (17 mg, 0.43 mmol) in Et_2_O (1.0 mL), a suspension of AlCl_3_ (18 mg, 0.14 mmol) in Et_2_O (1 mL) was added at 0 °C, and the whole was stirred at 0 °C for 30 min. A solution of **38** (49 mg, 0.14 mmol) in Et_2_O (1 mL) was added at −60 °C, and the whole was stirred at −60 °C for 21 h. Saturated aqueous NH_4_Cl (5 mL) was added. The whole was filtered through a pad of Celite, and the precipitate was washed with AcOEt (5 mL × 1 mL). The filtrate was extracted with AcOEt (3 mL × 10 mL). Combined organic layer was washed with H_2_O (1 mL × 10 mL) and brine (1 mL × 10 mL) and was dried over Na_2_SO_4_. Solvent was evaporated in vacuo, and the residue was purified by CC (hexane–AcOEt = 72:28–51:49) to give **39** as a yellow oil (25 mg, 50%) and **26** as a yellow oil (5 mg, 11%). **39**: IR (ATR) ν_max_ cm^−1^: 3413 (O-H). ^1^H-NMR (400 MHz, CDCl_3_) δ: 1.69 (3H, d, *J* = 6.9 Hz, C1-C*H*_3_), 1.92 (2H, dt, *J* = 6.4, 5.5 Hz, C1′-*H*_2_), 2.86 (1H, ddd, *J* = 16.5, 4.1, 0.9 Hz, one of C4-*H*_2_), 2.93 (1H, ddd, *J* = 16.5, 10.5, 1.4 Hz, one of C4-*H*_2_), 3.57 (3H, s, OC*H*_3_), 3.60 (3H, s, OC*H*_3_), 3.90 (2H, t, *J* = 5.5 Hz, C2′-*H*), 4.27 (1H, dtd, *J* = 10.5, 6.4, 4.1 Hz, C3-*H*), 5.01 (1H, d, *J* = 6.4 Hz, one of C10-OC*H*_2_O), 5.23 (1H, d, *J* = 6.4 Hz, one of C10-OC*H*_2_O), 5.28 (1H, d, *J* = 12.4 Hz, one of C9-OC*H*_2_O), 5.30 (1H, d, *J* = 12.4 Hz, one of C9-OC*H*_2_O), 5.39 (1H, q, *J* = 6.9 Hz, C1-*H*), 7.06 (1H, dd, *J* = 7.8, 0.9 Hz, C8-*H*), 7.30 (1H, dd, *J* = 8.2, 7.8 Hz, C7-*H*), 7.35 (1H, s, C5-*H*), 7.40 (1H, dd, *J* = 8.2, 0.9 Hz, C6-*H*). ^13^C-NMR (100 MHz, CDCl_3_) δ: 20.4, 34.4, 37.7, 56.5, 57.6, 61.4, 67.1, 69.4, 96.1, 101.7, 110.8, 118.8, 122.13, 123.7, 125.7, 130.3, 132.2, 136.0, 148.8, 152.5. HRESIMS *m/z* 363.1801 (calcd for C_20_H_27_O_6_: 363.1808). [α${]}_{D}^{25}$-196 (*c* 0.5, CHCl_3_).

*(1R,3S)-{9,10-Bis(methoxymethoxy)-3,4-dihydro-1-methyl-1H-naphtho[2,3-c]pyran-3-yl}acetic acid (**40**).* To a solution of **39** (16 mg, 0.043 mmol) in CH_2_Cl_2_ (0.2 mL), PIDA (21 mg, 0.064 mmol) was added at rt. After 5 min, TEMPO (1 mg, 0.0064 mmol) was added at rt, and the whole was stirred at rt for 14 h. Then, 5% aqueous Na_2_S_2_O_3_ (1.5 mL) was added, and the whole was extracted with CHCl_3_ (4 mL × 5 mL). Combined organic layer was washed with brine (1 mL × 5 mL) and dried over Na_2_SO_4_. Solvent was evaporated in vacuo, to give a yellow oil (19 mg). The residue was dissolved in acetone (0.25 mL), *t*-BuOH (0.51 mL), and H_2_O (0.12 mL). 2-Methyl-2-butene (0.13 mL, 1.23 mmol), NaH_2_PO_4_·2H_2_O (29 mg, 0.19 mmol), and NaClO_2_ (10 mg, 0.11 mmol) was successively added in each 10 min, and the whole was stirred at rt for 1 h. 5% HCl (0.3 mL) was added, and the whole was extracted with CHCl_3_ (4 mL × 5 mL). Combined organic layer was washed with brine (1 mL × 5 mL) and dried over Na_2_SO_4_. Solvent was evaporated in vacuo, and the residue was purified by CC (CHCl_3_–MeOH = 60:40) to give **40** as a yellow oil (11 mg, 65%). IR (ATR) ν_max_ cm^−1^: 3500–2800 (O-H), 1711 (C=O). ^1^H-NMR (400 MHz, CDCl_3_) δ: 1.68 (3H, d, *J* = 6.9 Hz, C1-C*H*_3_), 2.70 (2H, brs, C4-*H*_2_), 2.85–2.98 (2H, m, C1′-*H*), 3.56 (3H, s, OC*H*_3_), 3.59 (3H, s, OC*H*_3_), 4.48 (1H, brs, C3-*H*), 4.99 (1H, d, *J* = 6.4 Hz, one of C10-OC*H*_2_O), 5.21 (1H, d, *J* = 6.4 Hz, one of C10-OC*H*_2_O), 5.27 (1H, d, *J* = 11.4 Hz, one of C9-OC*H*_2_O), 5.29 (1H, d, *J* = 11.4 Hz, one of C9-OC*H*_2_O), 5.59 (1H, q, *J* = 6.4 Hz, C1-*H*), 7.05 (1H, d, *J* = 7.8 Hz, C8-*H*), 7.28 (1H, dd, *J* = 7.8, 7.8 Hz, C7-*H*), 7.33 (1H, s, C5-*H*), 7.37 (1H, d, *J* = 7.8 Hz, C6-*H*). ^13^C-NMR (100 MHz, CDCl_3_) δ: 20.1, 33.7, 41.0, 56.5, 57.5, 63.8, 69.8, 96.0, 101.7, 110.8, 118.9, 122.2, 123.8, 125.8, 130.0, 131.4, 136.0, 148.8, 152.5, 175.3. HRESIMS *m/z* 377.1616 (calcd for C_20_H_25_O_7_: 377.1600). [α${]}_{D}^{25}$ − 144.0 (*c* 1.1, CHCl_3_).

*(1R,3S)-(3,4-Dihydro-10-hydroxy-9-methoxymethoxy-1-methyl-1H-naphtho[2,3-c]pyran-3-yl)acetic acid (**41**) and (1R,3S)-(3,4-Dihydro-9,10-dihydroxy-1-methyl-1H-naphtho[2,3-c]pyran-3-yl)acetic acid (DDHK, **3**).* To a solution of **40** (23 mg, 0.061 mmol) in THF (1 mL), 6 M HCl (0.1 mL) was added, and the whole was stirred at 50 °C for 20 min. After cooling to rt, CH_2_Cl_2_ (3 mL) was added, and the whole was washed with H_2_O (1 mL × 2 mL). Aqueous layer was extracted with CH_2_Cl_2_ (1 mL × 3 mL). The combined organic layer was washed with brine (1 mL × 2 mL) and dried over Na_2_SO_4_. Solvent was evaporated in vacuo, and the residue was purified by CC (CHCl_3_–MeOH = 97:3–90:10) to give **41** as a brown oil (14 mg. 71%) and **3** as a brown oil (5 mg, 27%). **41**: IR (ATR) ν_max_ cm^−1^: 3390 (O-H), 1709 (C=O). ^1^H-NMR (400 MHz, CDCl_3_) δ: 1.65 (3H, d, *J* = 6.6 Hz, C1-C*H*_3_), 2.73 (2H, d, *J* = 6.2 Hz, two of C4 or C1′-*H*_2_), 2.87 (1H, dd, *J* = 15.8, 10.5 Hz, one of C4 or C1′-*H*_2_), 2.96 (1H, dd, *J* = 16.4, 3.2 Hz, one of C4 or C1′-*H*_2_), 3.59 (3H, s, OCH_3_), 4.47–4.54 (1H, m, C3-*H*), 5.43 (1H, q, *J* = 6.4 Hz, C1-*H*), 5.44 (2H, s, C9-OC*H*_2_O), 6.98 (1H, d, *J* = 7.3 Hz, C8-*H*), 7.09 (1H, s, C5-*H*), 7.24 (1H, dd, *J* = 8.2, 7.8 Hz, C7-*H*), 7.36 (1H, d, *J* = 8.2 Hz, C6-*H*), 9.56 (1H, s, O*H*). ^13^C-NMR (100 MHz, CDCl_3_) δ: 18.9, 33.9, 40.5, 56.9, 63.9, 69.5, 95.8, 107.2, 113.7, 117.8, 120.6, 122.1, 125.6, 132.0, 135.1, 149.1, 153.6, 173.1. HRESIMS *m/z* 333.1335 (calcd. for C_18_H_21_O_6_: 333.1338). [α${]}_{D}^{25}$ + 10.6 (*c* 0.3, CHCl_3_). **3**: IR (ATR) ν_max_ cm^−1^: 2924 (O-H), 1712 (C=O). ^1^H-NMR (400 MHz, CD_3_OD) δ: 1.49 (3H, d, *J* = 6.4 Hz, C1-C*H*_3_), 2.45 (1H, dd, *J* = 15.3, 8.2 Hz, one of C4-*H*_2_), 2.52 (1H, dd, *J* = 15.3, 4.8 Hz, C4-*H*_2_), 2.65 (1H, dd, *J* = 15.8, 10.1 Hz, one of C1′-*H*_2_), 2.84 (1H, dd, *J* = 15.8, 3.2 Hz, one of C1′-*H*_2_), 4.34–4.43 (1H, m, C3-*H*), 5.16 (1H, q, *J* = 6.6 Hz, C1-*H*), 6.53 (1H, dd, *J* = 7.6, 0.9 Hz, C8-*H*), 6.92 (1H, s, C5-*H*), 7.03 (1H, dd, *J* = 8.2, 7.6 Hz, C7-*H*), 7.09 (1H, d, *J* = 8.2 Hz, C6-*H*). ^13^C-NMR (125 MHz, CD_3_OD) δ: 19.4, 35.1, 42.0, 65.7, 70.2, 108.2, 114.6, 120.6, 120.8, 126.8, 133.7, 136.9, 151.0, 154.6, 174.9. HRESIMS *m/z* 289.1082 (calcd for C_16_H_17_O*5*: 289.1076). [α${]}_{D}^{25}$ + 30.9 (*c* 0.2, MeOH).

*Transformation of MOM ether **41** to DDHK (**3**).* To a solution of **41** (5 mg, 0.016 mmol) in CH_2_Cl_2_ (0.4 mL), BCl_3_ (1.0 M in CH_2_Cl_2_, 0.05 mL, 0.05 mmol) was added at −78 °C, and the mixture was stirred at −78 °C for 45 min. H_2_O (1.3 mL) was added, and the whole was extracted with AcOEt (1 mL × 10 mL, 3 mL × 5 mL). The combined organic layer was washed with brine (1 mL × 5 mL) and was dried over Na_2_SO_4_. The solvent was evaporated in vacuo, and the residue was purified by CC (CHCl_3_–MeOH = 9:1) to give **3** as a yellow oil (3 mg, 69%). The physical properties of **3** obtained here were identical with those prepared as described above.

*Semisynthesis of DDHK (****3****) and epi-DDHK (****7****) from (S)-DNPA (****6****).* To a solution of (S)-DNPA (**6**) (2 mg, 6.3 x 10^–3^ mmol) in MeOH (0.2 mL), NaBH_4_ (2.5 mg, 6.8 × 10^−2^ mmol) was added at 0 °C, and the mixture was stirred at 0 °C for 10 min. The solvent was evaporated in vacuo, and the residue was partitioned with AcOEt (1 mL) and 10% HCl (0.5 mL). Aqueous layer was extracted with AcOEt (2 mL × 1 mL). Combined organic layer was washed with brine (1 mL × 1 mL) and dried over Na_2_SO_4_. The solvent was evaporated in vacuo. The residue was purified by RP HPLC (Cosmosil AR-II, 26 mm × 250 mm, CH_3_OH-H_2_O 2:1, 2 mL/min) to give DDHK (**3**) (0.5 mg, 28%) and *epi*-DDHK (**7**) (0.4 mg, 22%).

## 4. Conclusions

In conclusion, we have accomplished the first total synthesis of DDHK (**3**) and its epimer *epi*-DDHK (**7**). Et_3_SiH reduction of tricyclic hemiacetal **22** afforded benzoisochromane **23** with 1,3-*cis* stereochemistry, which was converted to *epi*-DDHK (**7**) through an oxidation/deprotection sequence. Although several trials using silane reagents did not deliver any reduction products, stereoselective reduction was further examined for bicyclic acetal **38**. AlH_3_ reduction of the bis-MOM-protected bicyclic acetal **38** yielded the 1,3-*trans* isomer **39** as the major product, which was finally transformed to DDHK (**3**). In addition, DDHK (**3**) and *epi*-DDHK (**7**) were accessed by a semisynthetic approach based on the reduction of (*S*)-DNPA (**6**), and the obtained products were identified by comparison of their spectral data with those of the synthetic compounds. The semisynthetic DDHK (**3**) obtained by this method has been applied to study the biosynthesis of ACT (**1**) and clarify the long-standing ambiguity regarding the role of the converting enzyme system ActVA-ORF5/ActVB in the C-6 and C-8 hydroxylation [3] of DDHK (**3**) by in vitro enzymatic analysis [5]. These compounds are also anticipated to serve as useful reference samples for preparing stereoisomers of DDHK from (*R*)-DNPA, a biosynthetic precursor of nanaomycin A (**9**), and they are expected to enable further elucidation of the biosynthetic pathways of other BIQ antibiotics with different stereochemical configurations [35,36].

## Figures and Tables

**Table 1:**
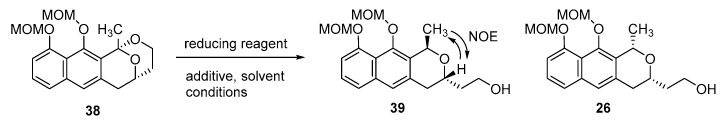
Diastereoselective reduction trials of bicyclic acetal **38** to *trans* (**39**) and *cis* (**26**) benzoisochromanes.

Run	[H^−^] (eq)	Additive (eq)	Solvent	Conditions	39/26	Isolated Yield (%)	
						39	26
1	NaBH_3_CN (4.4)	10% HCl (1.2)	CH_3_OH	rt, 15 min	19:81	13	69
2	BH_3_THF (4.0)	none	THF	−45 °C, 2 h; −20 °C, 1 h; rt, 41 h	26:74	-	-
3	DIBAL-H (5)	none	CH_2_Cl_2_	−78 °C, 3.5 h	53:47	-	-
4	DIBAL-H (5)	none	Et_2_O	−78 °C, 30 h	8:92	-	-
5	DIBAL-H (10)	none	THF	−45 °C, 71 h; −20 °C, 20 h	61:39	55	26
6	AlH_3_ (4)	none	Et_2_O	−78 °C, 28 h	76:24	10	3
7	AlH_3_ (4)	none	Et_2_O	−60 °C, 21 h	75:25	50	11
8	AlH_3_ (4)	none	Et_2_O	−60 °C, 68 h	71:29	50	9

## Data Availability

Data are contained within the article or Supplementary Materials.

## Supplementary Materials

NMR spectra of synthesized compounds are available online.
