# Supplementary material for: Total Synthesis of 6-Deoxydihydrokalafungin, a Key Biosynthetic Precursor of Actinorhodin, and Its Epimer
Source: Molecules. 2021 Oct 22;26(21):6397. doi: 10.3390/molecules26216397 (PMC8587838; doi:10.3390/molecules26216397)
Supplement: Supplementary file 1 [file molecules-26-06397-s001.zip › molecules-1421515-supplementary.pdf]

Supplementary Materials

Total synthesis of 6-deoxydihydrokalafungin, a key biosynthetic precursor of actinorhodin, and its epimer

Takuya Kumamoto,[1,*] Mika Kainuma,[2] Azusa Takahashi,[2] Yoshika Matsuo,[2]
Kazuaki Katakawa,[2] Takaaki Taguchi,[3] and Koji Ichinose[2]

[1]*Department of Synthetic Organic Chemistry, Graduate School of Biomedical and Health Sciences, Hiroshima University, 1-2-3, Kasumi, Minami-ku, Hiroshima, 734-8553, Japan*

[2]*Research Institute of Pharmaceutical Sciences, Musashino University, 1-1-20, Shinmachi, Nishitokyo-shi, Tokyo 202-8585, Japan*

[3]*National Institute of Health Sciences, 3-25-26, Tonomachi, Kawasaki-ku, Kawasaki-shi, Kanagawa 210-9501, Japan*

tkum632@hiroshima-u.ac.jp

Table of Contents

1. Copies of ^{1}H and ^{13}C NMR Spectra 2

1. Copies of ^{1}H and ^{13}C NMR Spectra.

Compound **18**.

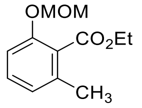

18

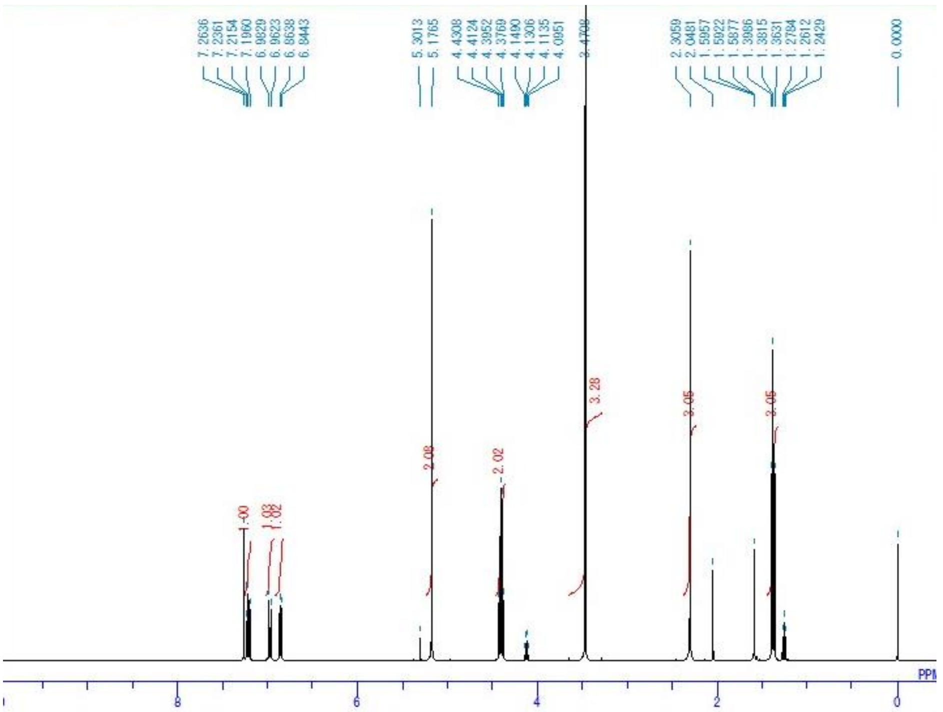

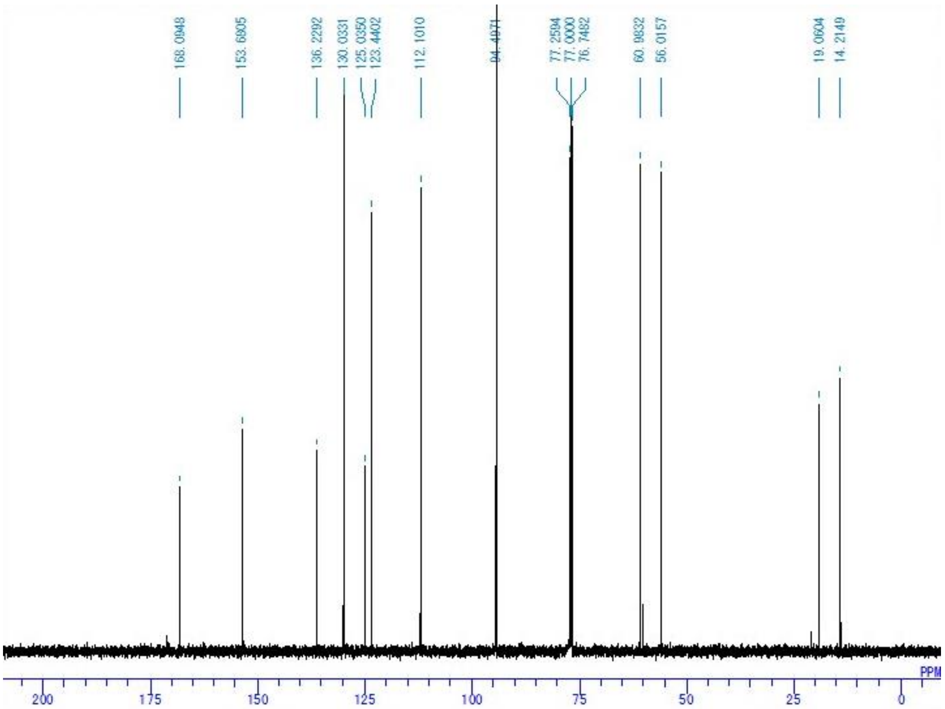

Compound **21**.

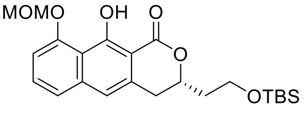

21

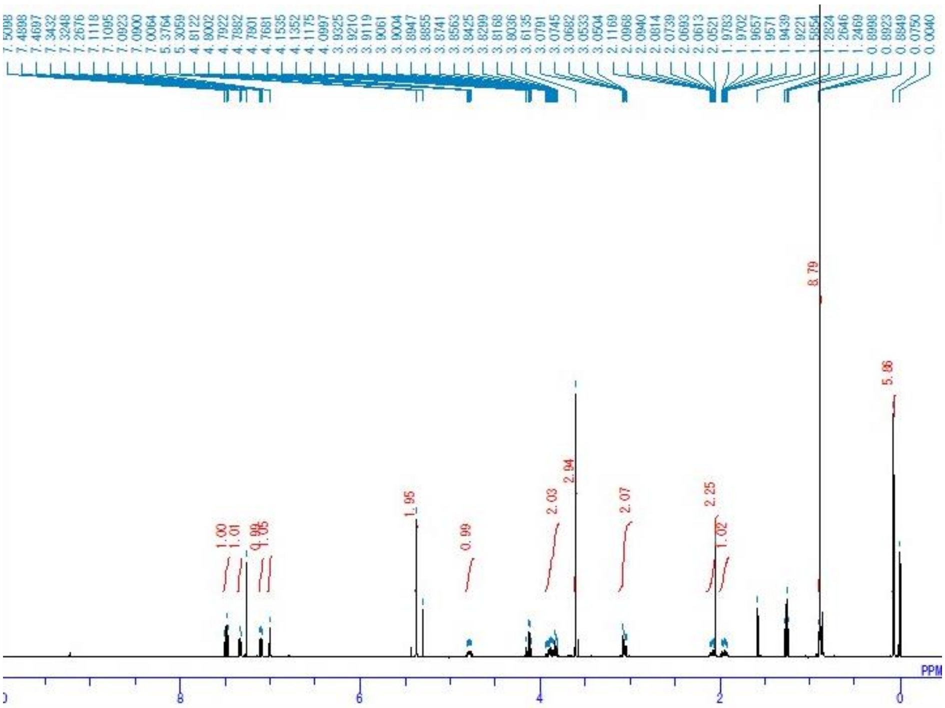

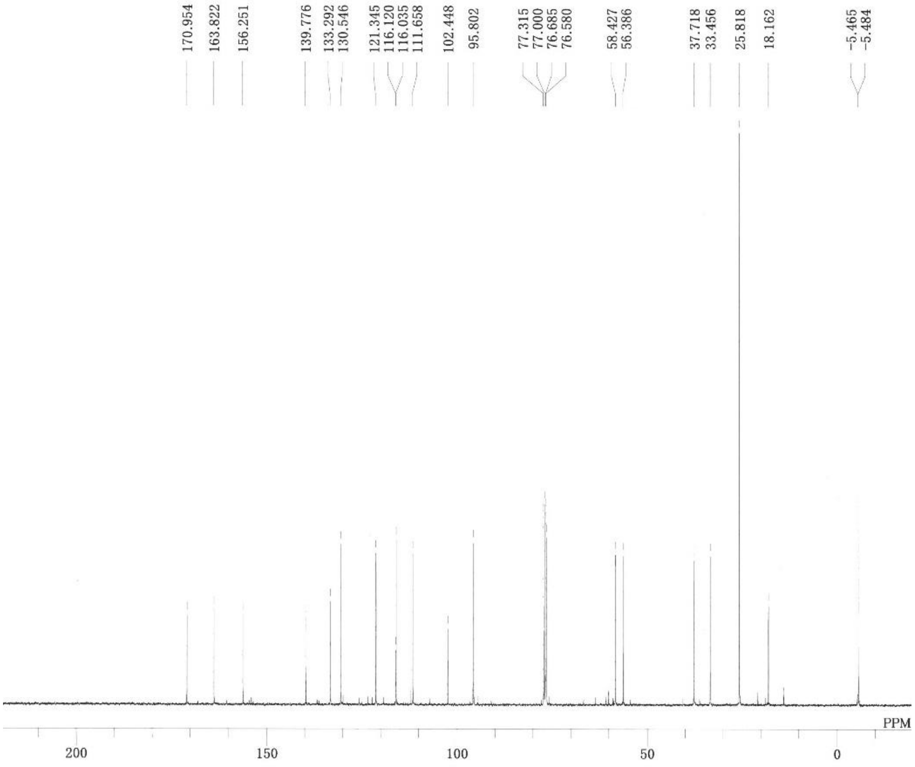

Compound **23**.

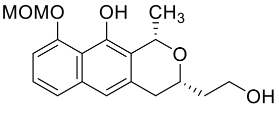

23

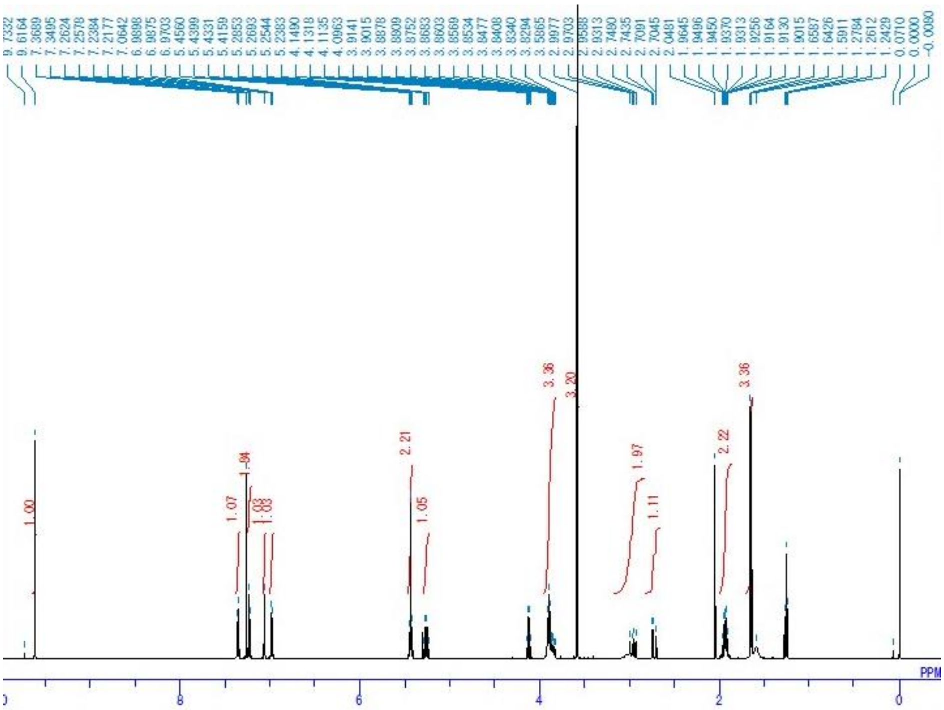

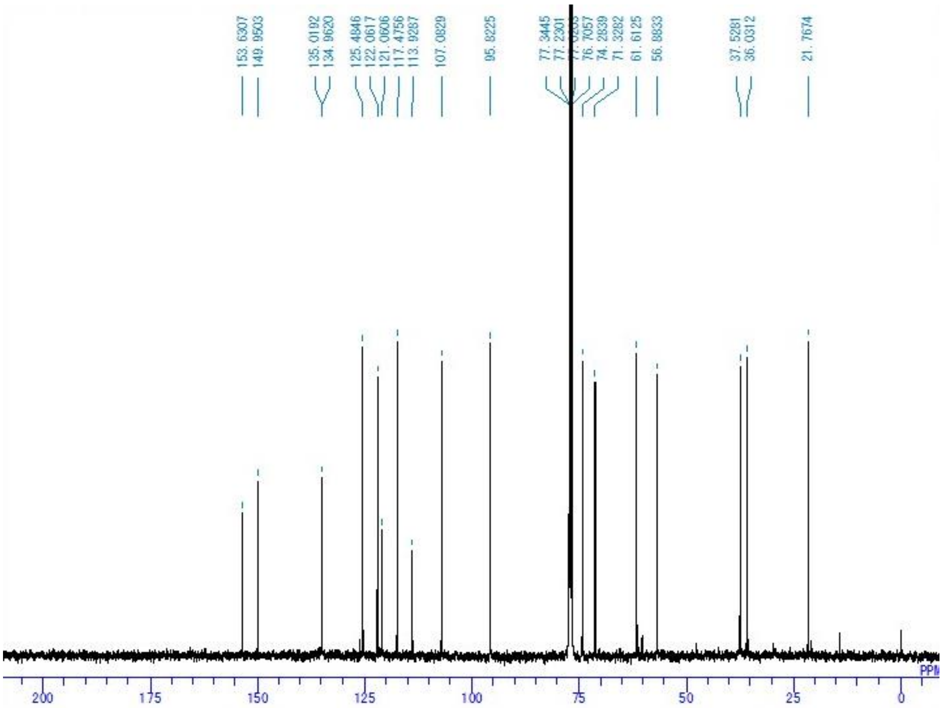

Compound **25**.

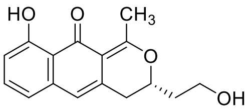

25

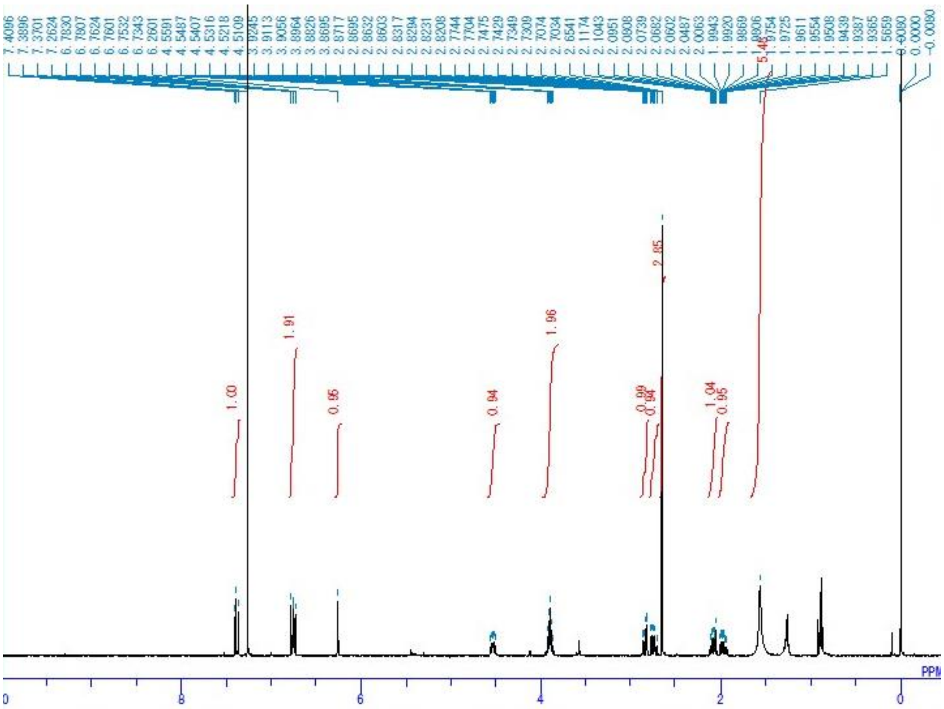

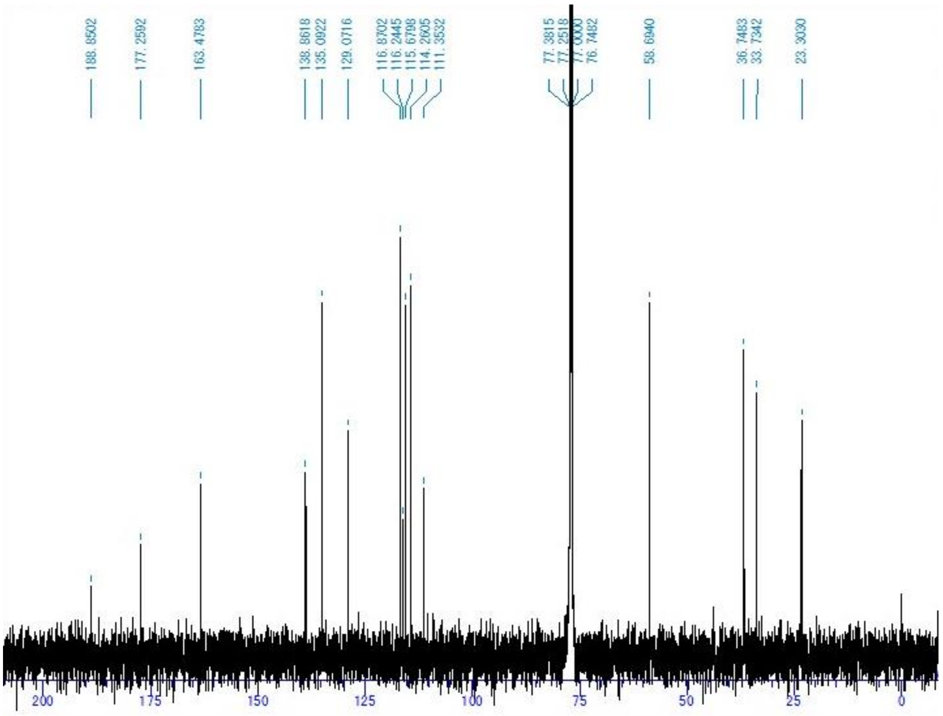

Compound **26**.

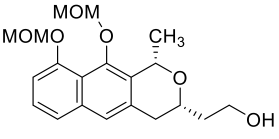

26

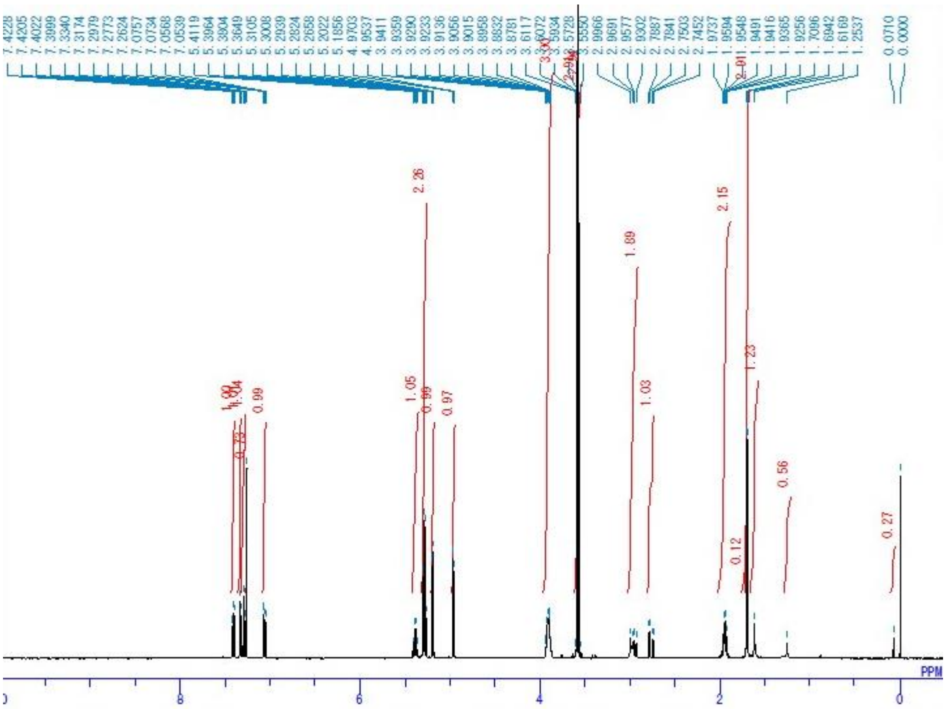

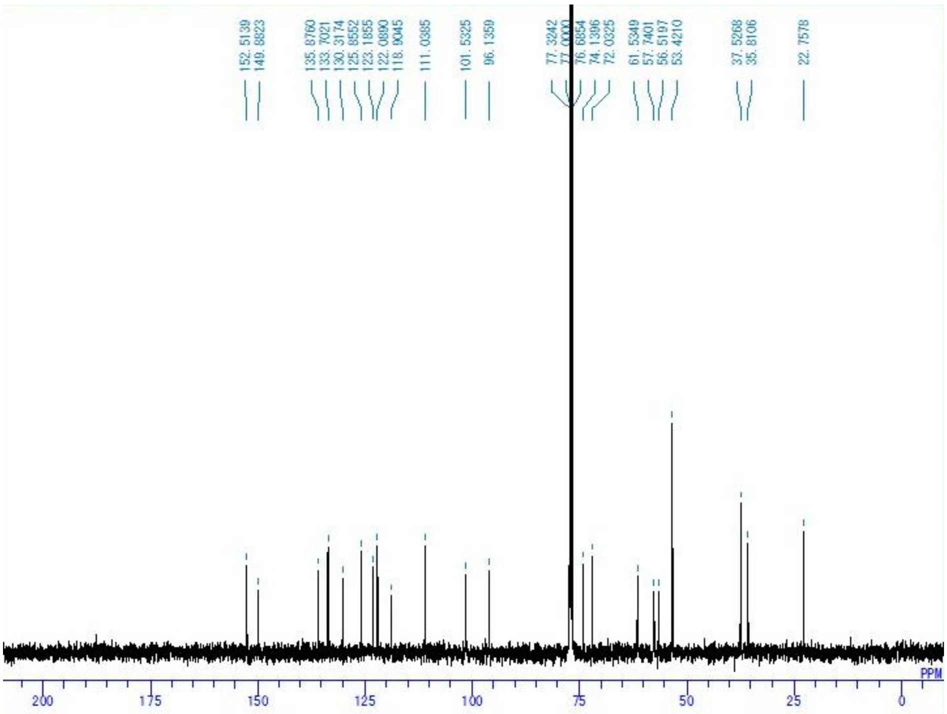

Compound **27**.

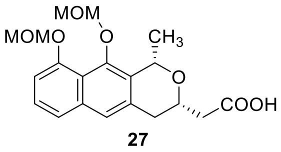

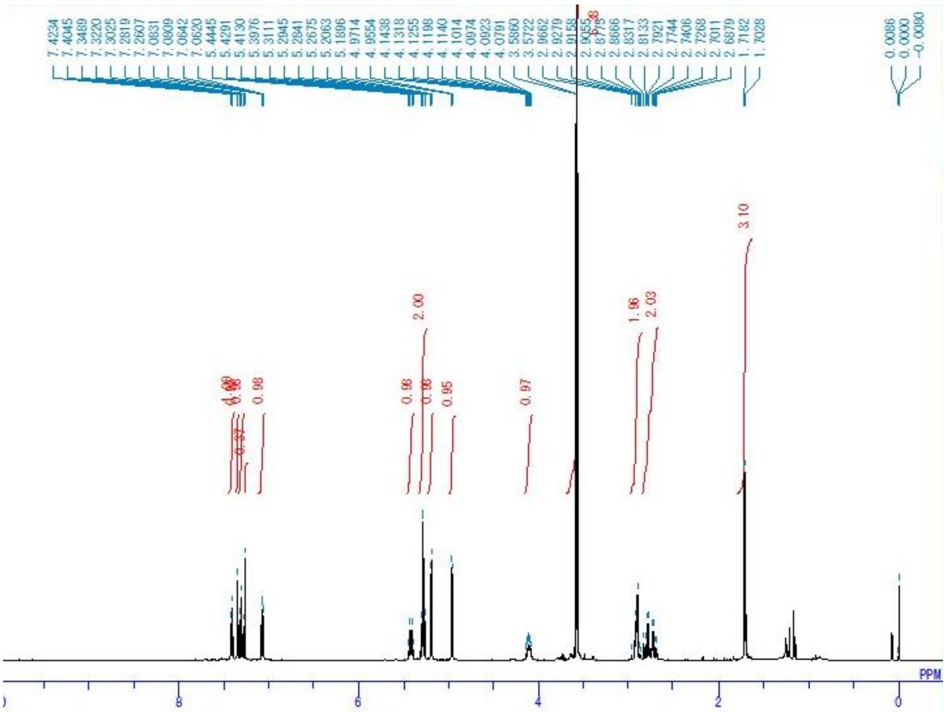

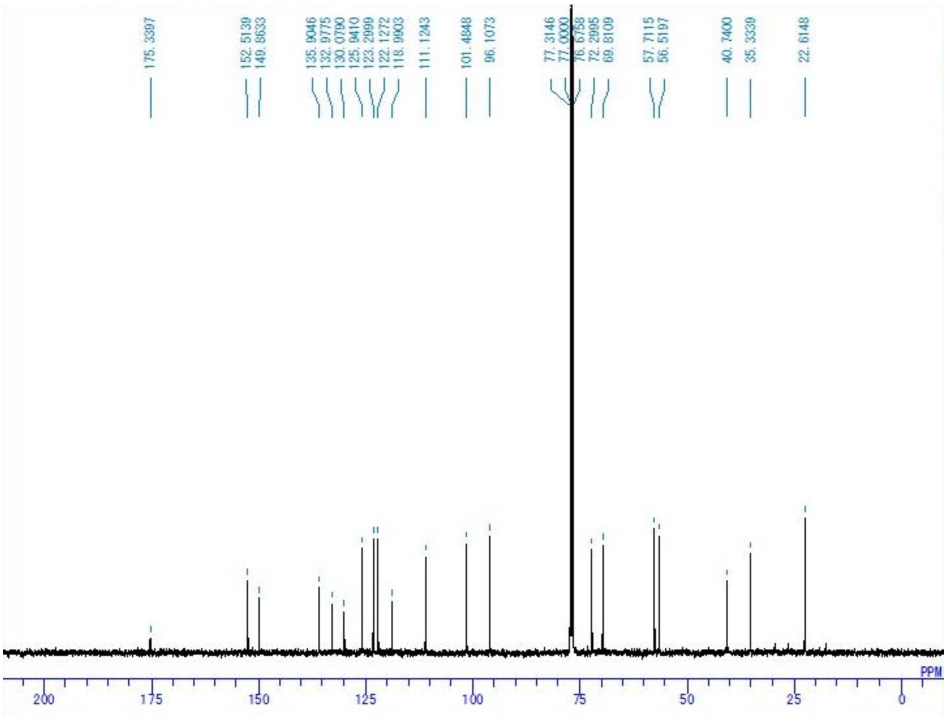

Compound **28**.

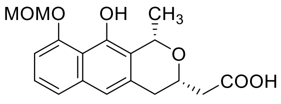

28

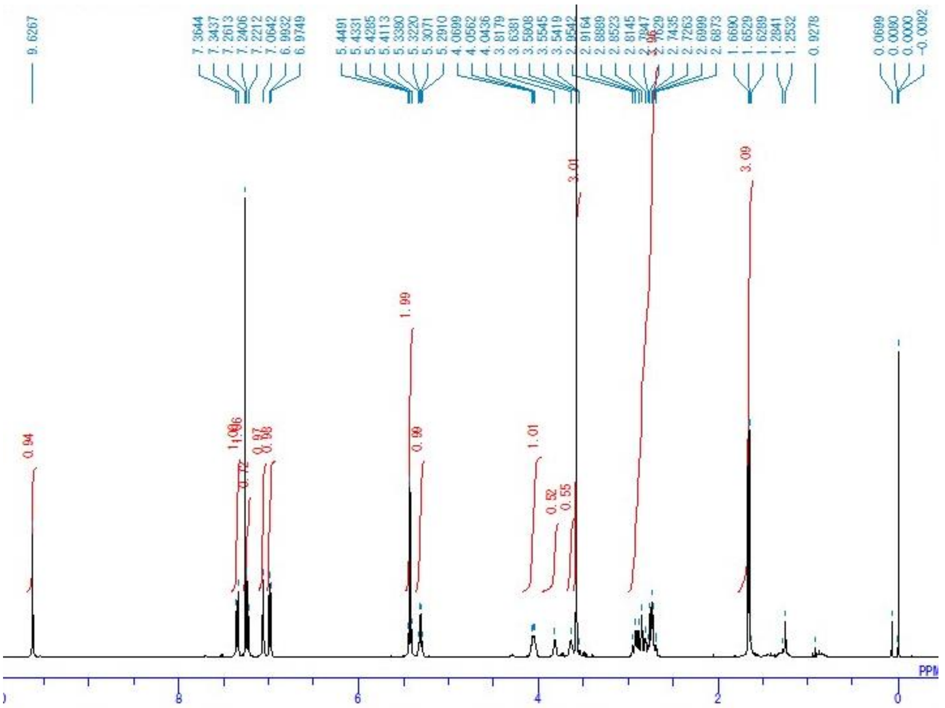

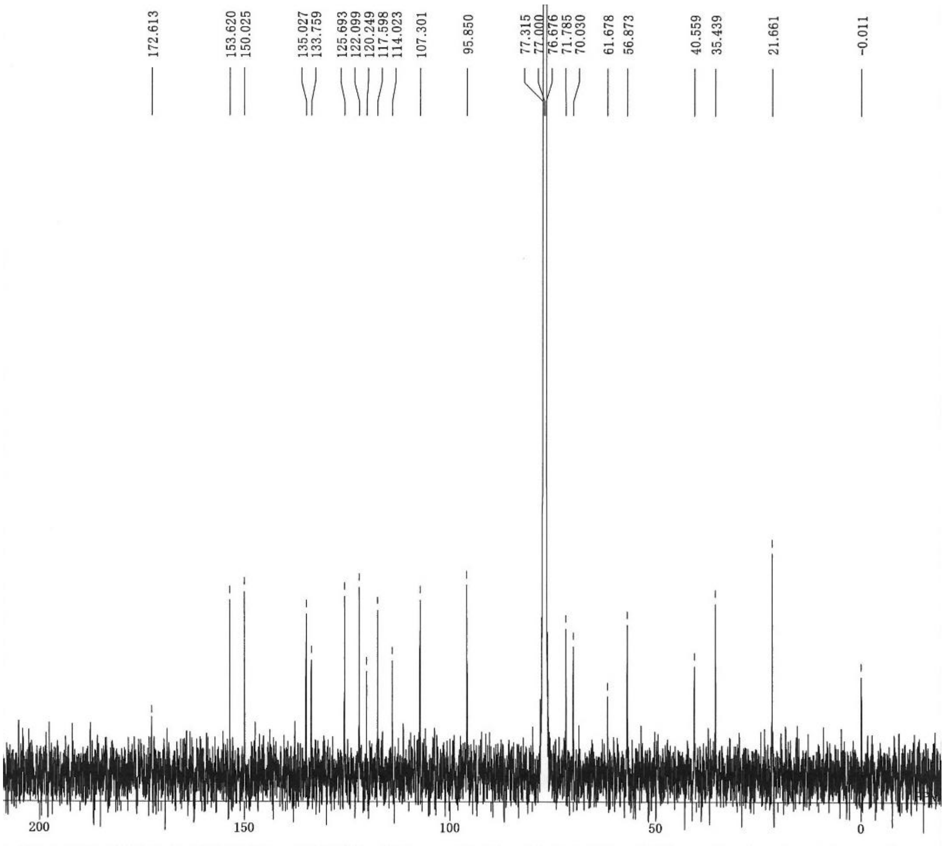

epi-DDHK (**7**).

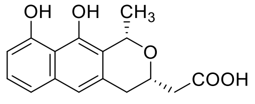

OH
OH
CH3
O
COOH

7

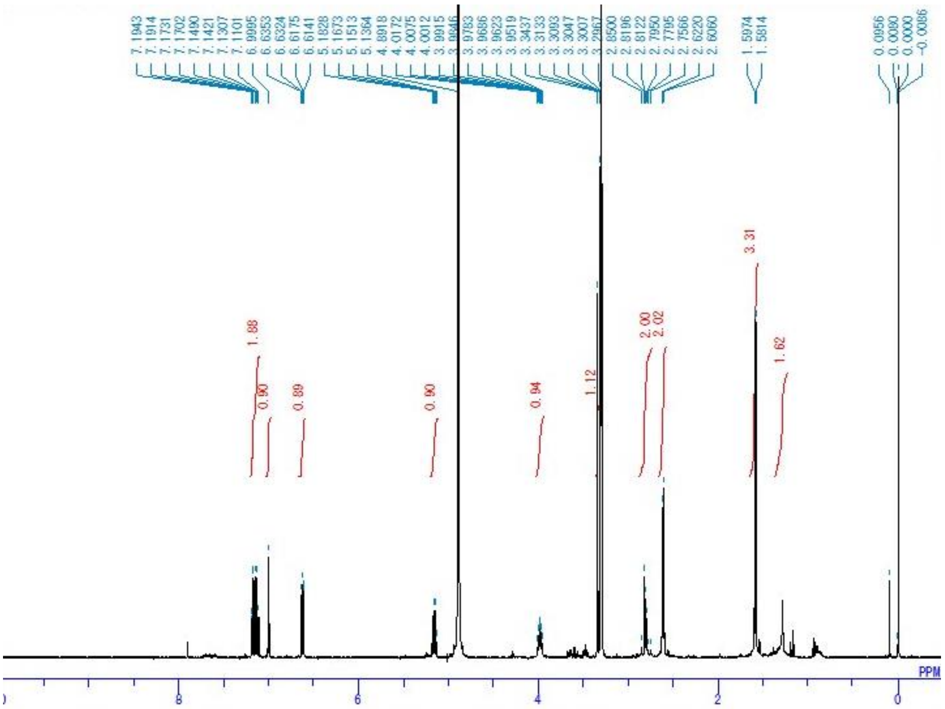

7.1943
7.1914
7.1731
7.1702
7.1490
7.1421
7.1307
7.1101
6.9995
6.6353
6.6324
6.6175
6.6141
5.1828
5.1673
5.1513
5.1364
4.8918
4.0172
4.0075
4.0012
3.9915
3.9846
3.9783
3.9686
3.9623
3.9519
3.3437
3.3133
3.3093
3.3047
3.3007
3.2967
2.8500
2.8196
2.8122
2.7950
2.7795
2.7566
2.6220
2.6060
1.5974
1.5814
0.0956
0.0090
0.0000
-0.0086
1.88
0.90
0.89
0.90
0.94
1.12
2.00
2.02
3.31
1.62
PPM
8
6
4
2
0

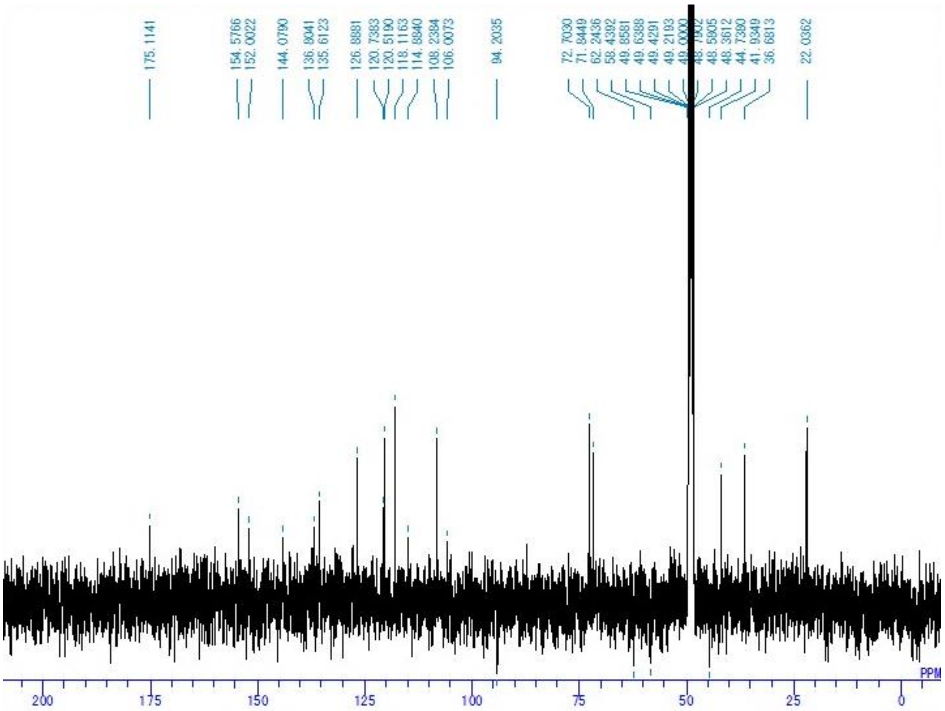

175.1141
154.5766
152.0022
144.0790
136.9041
135.6123
126.8881
120.7383
120.5190
118.1163
114.8840
108.2384
106.0073
94.2035
72.7030
71.8449
62.2436
58.4392
49.8581
49.6388
49.4291
49.2193
49.0000
48.7902
48.5805
48.3612
44.7380
41.9349
36.6813
22.0362
PPM
200
175
150
125
100
75
50
25
0

Compound **30**.

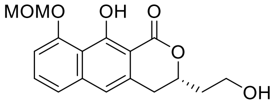

30

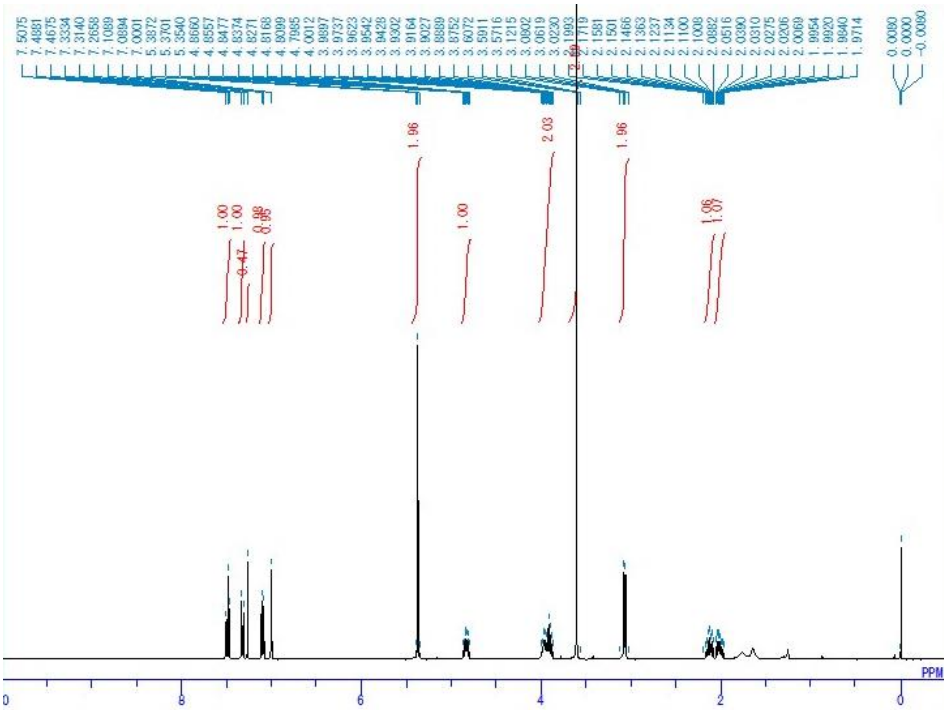

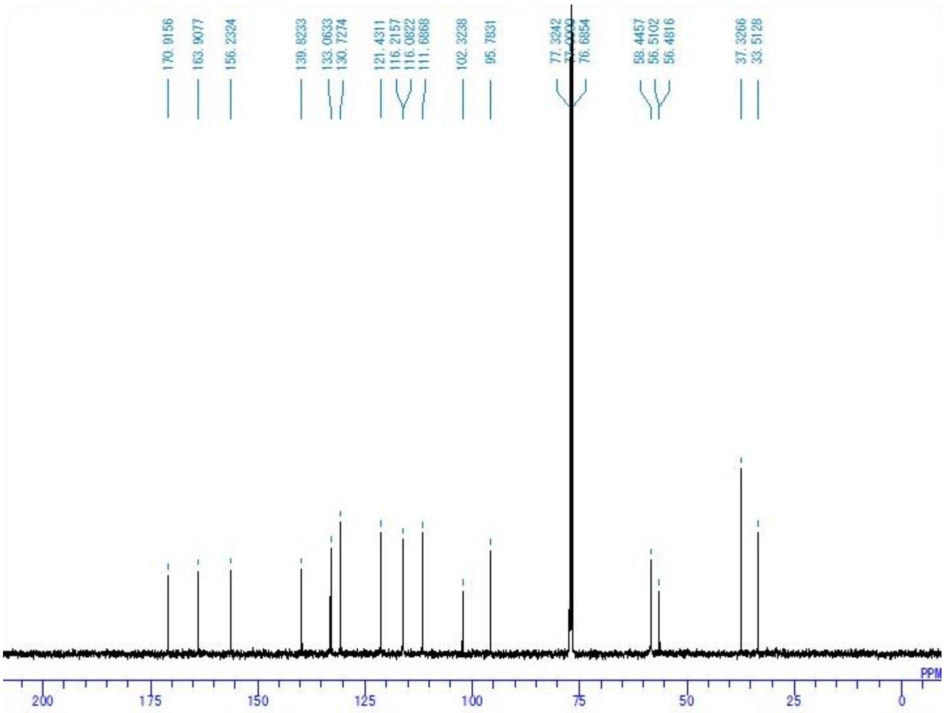

Compound **32**.

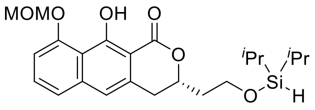

32

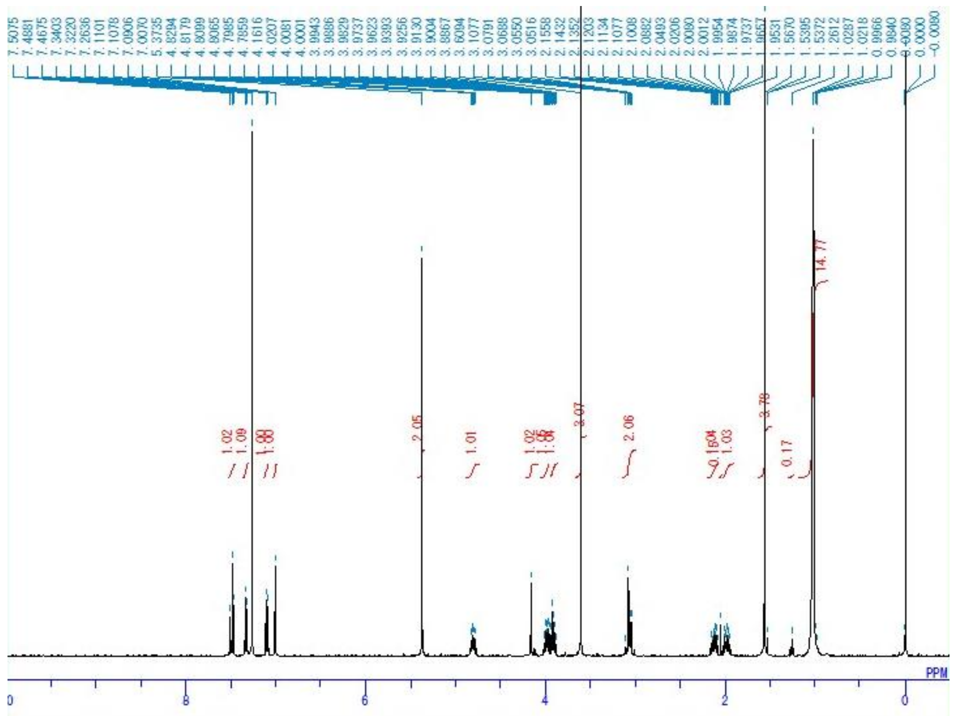

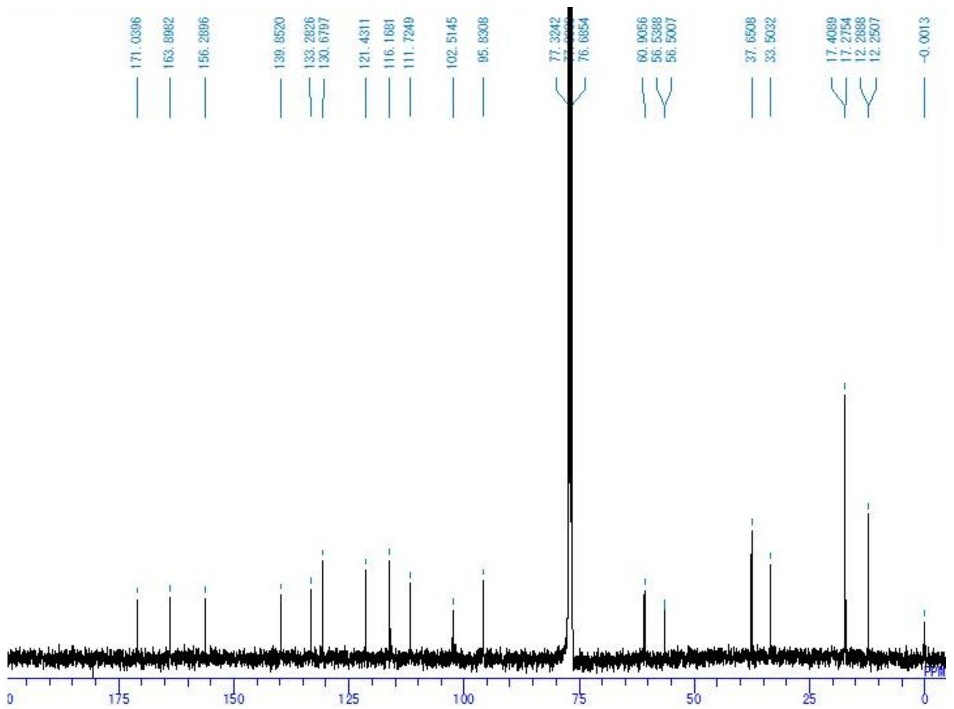

Compound **33**.

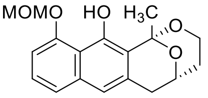

33

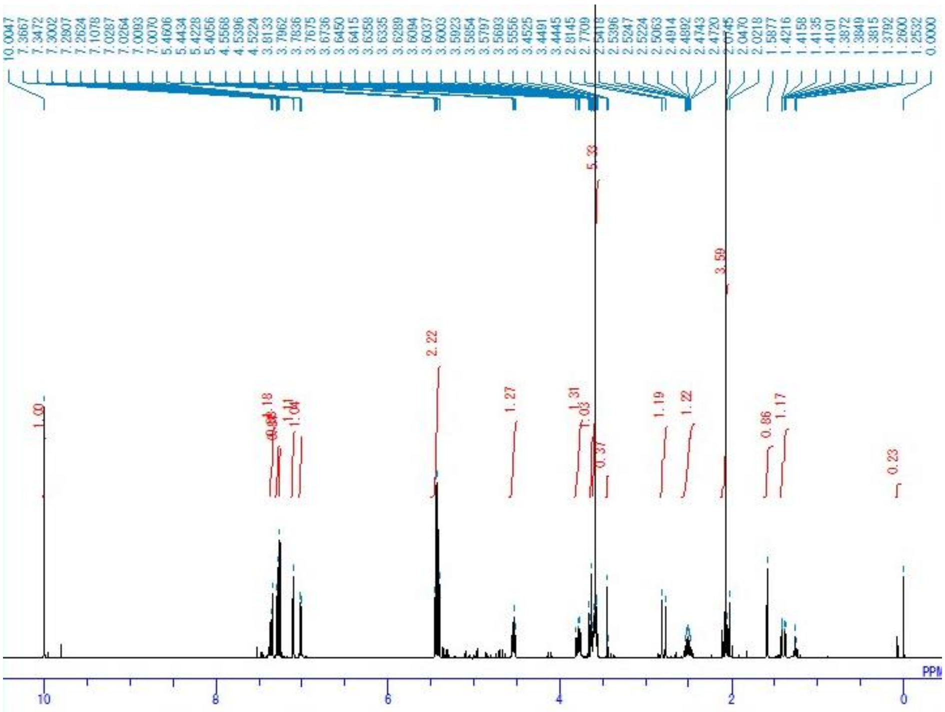

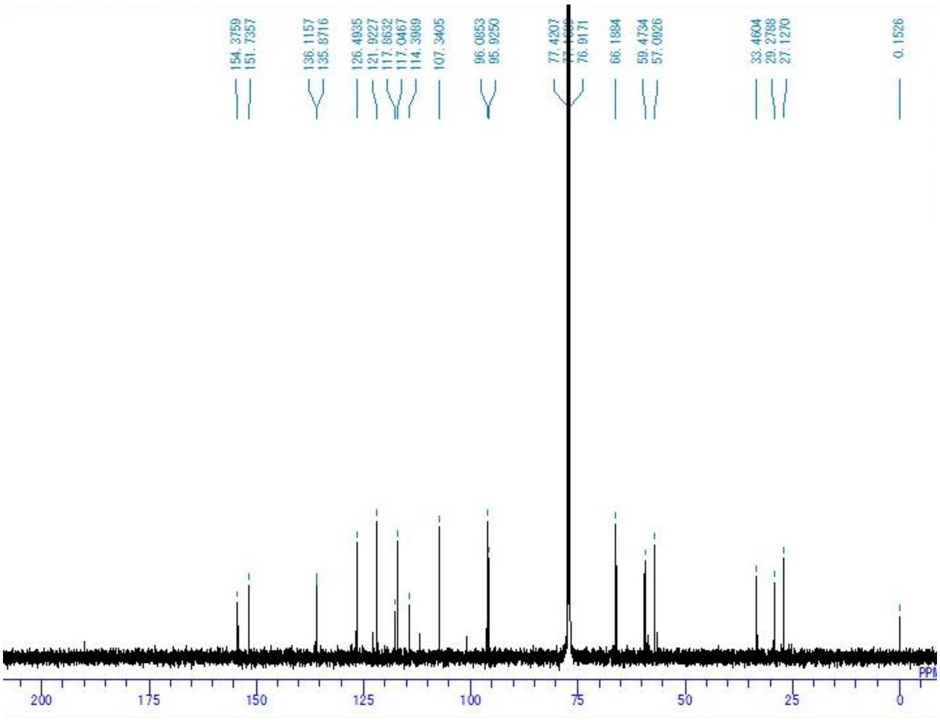

Compound **38**.

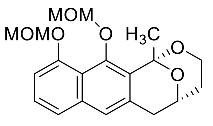

38

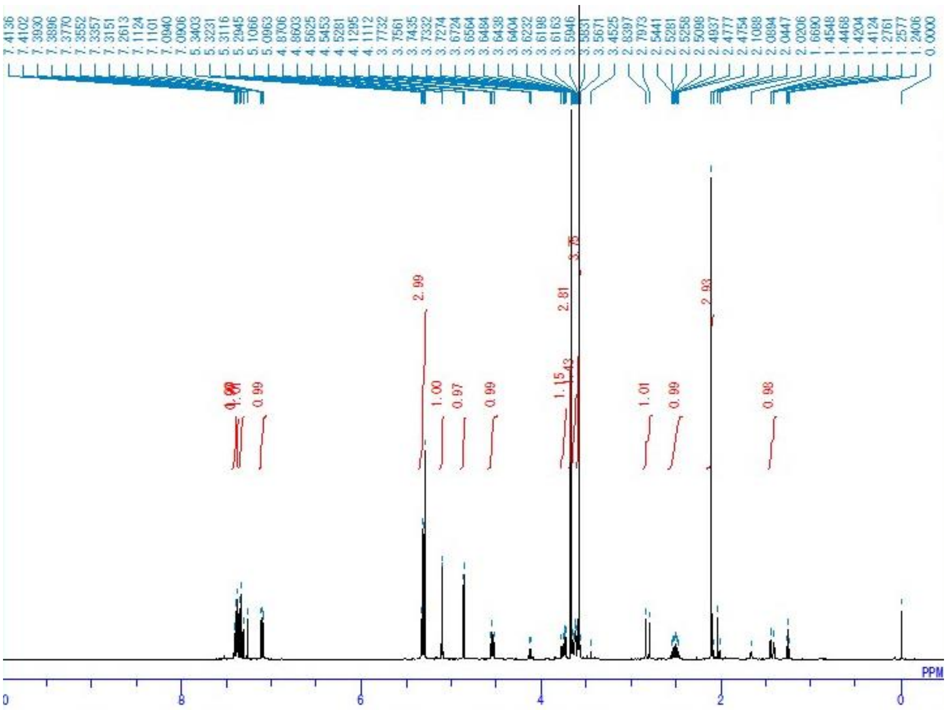

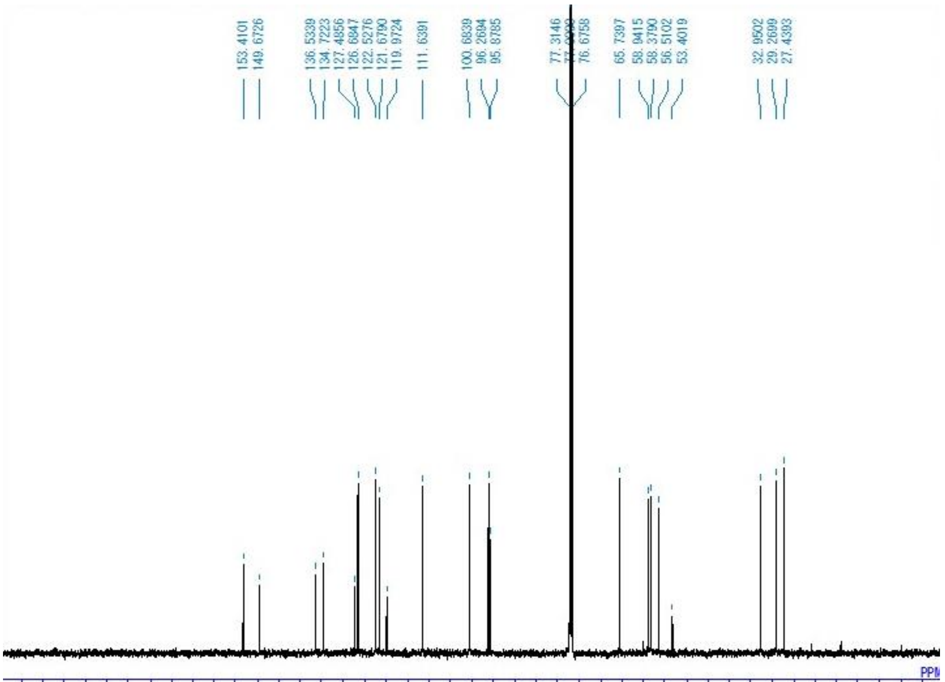

Compound **39**.

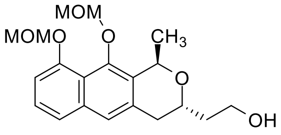

39

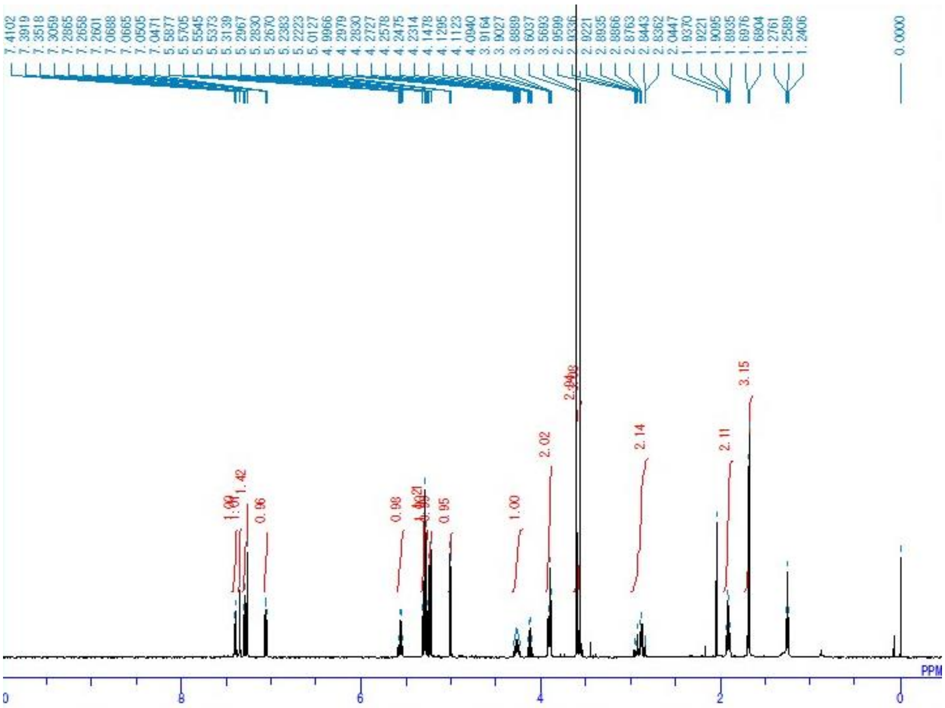

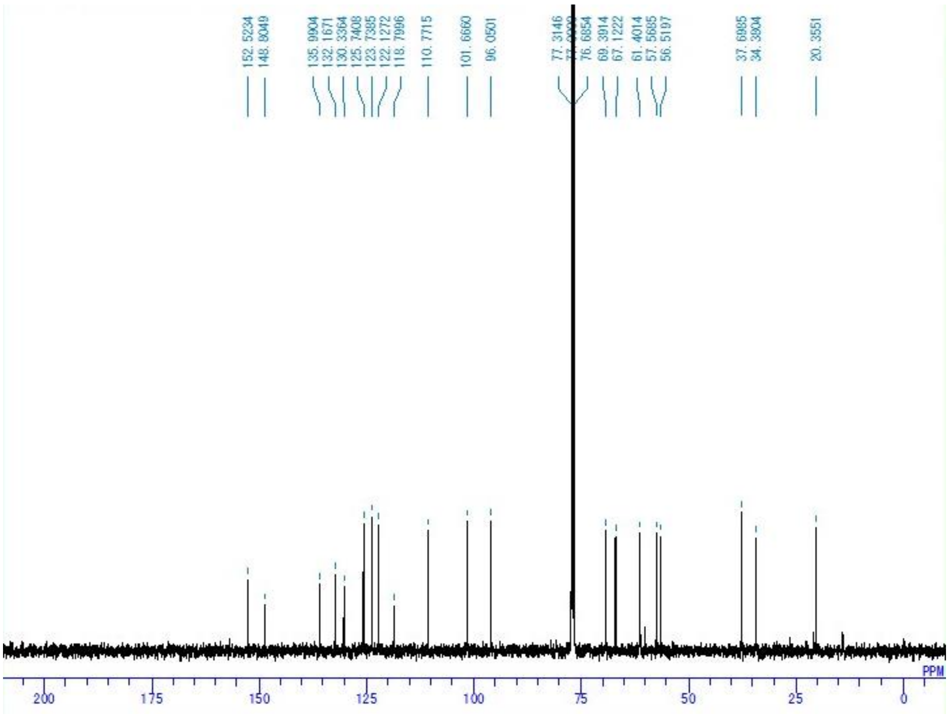

Charts of NOE experiments of compound **39**.

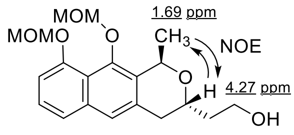

39

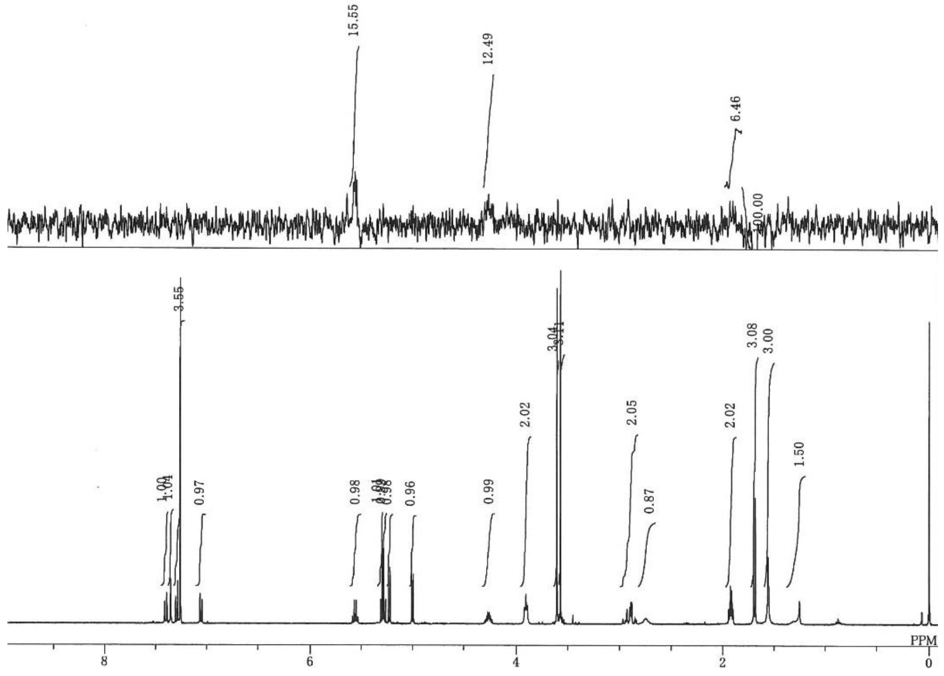

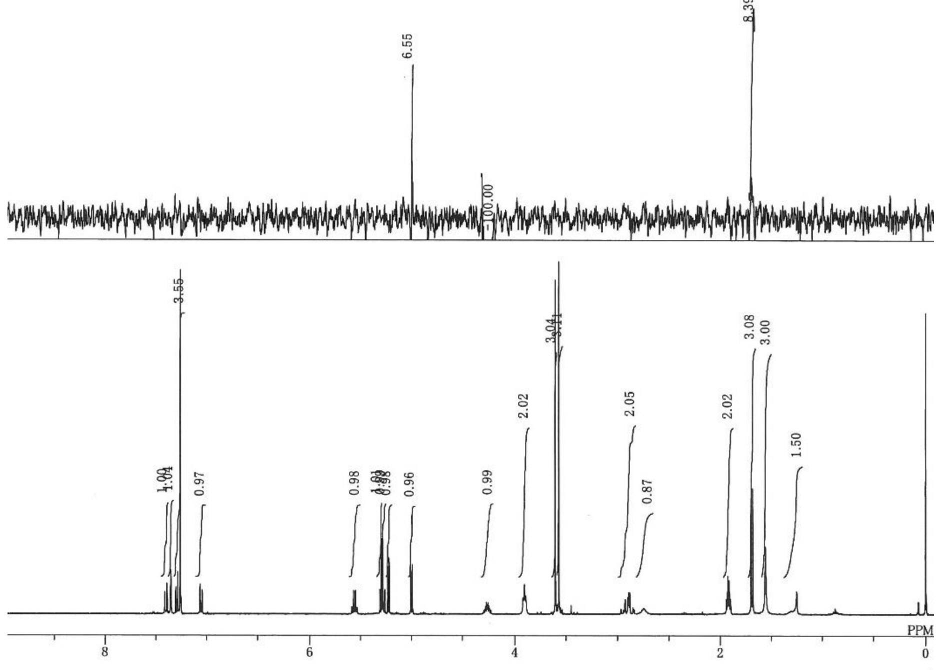

Compound **40**.

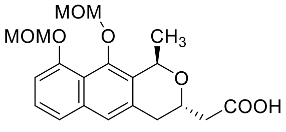

40

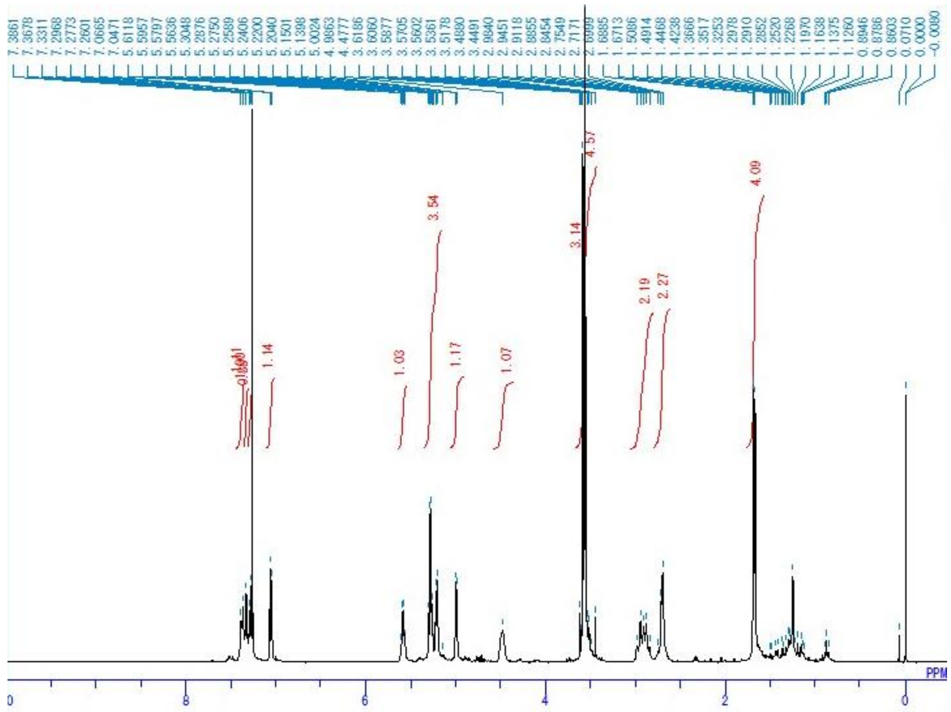

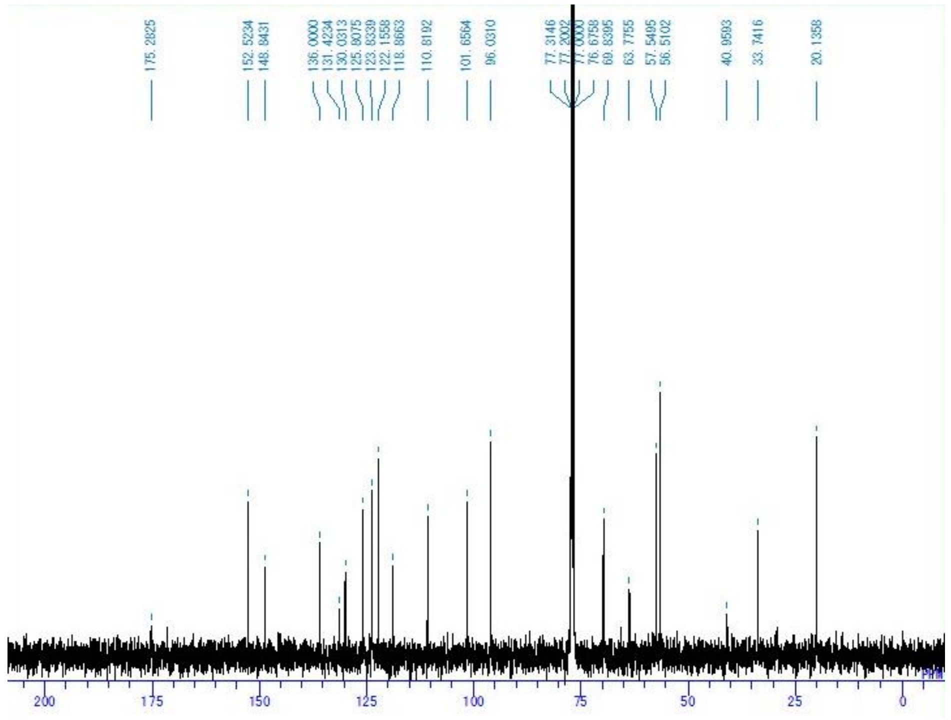

Compound **41**.

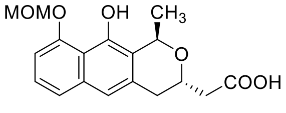

41

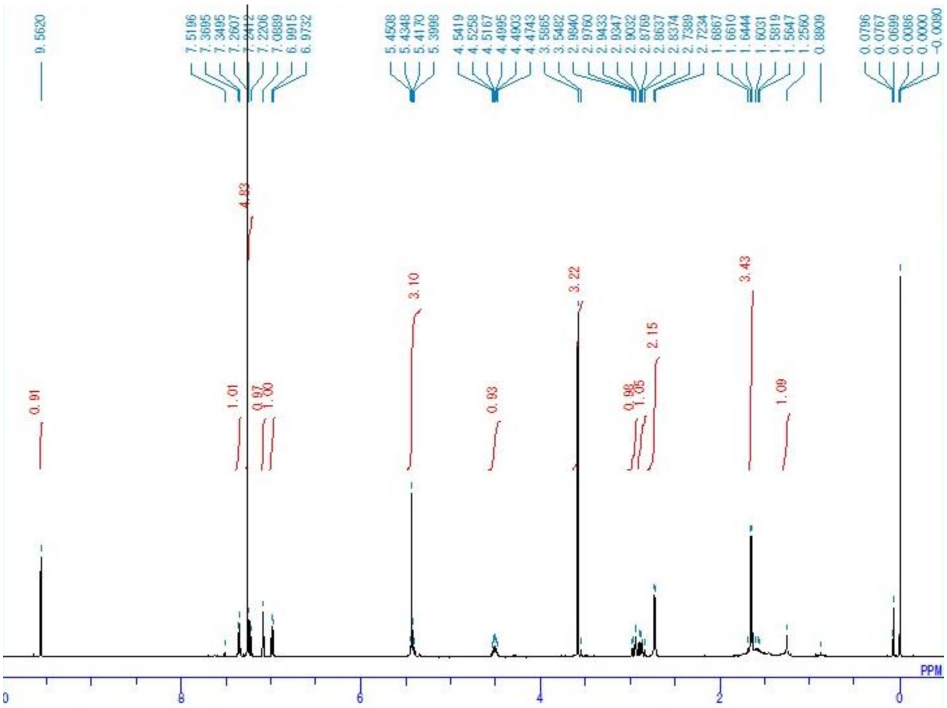

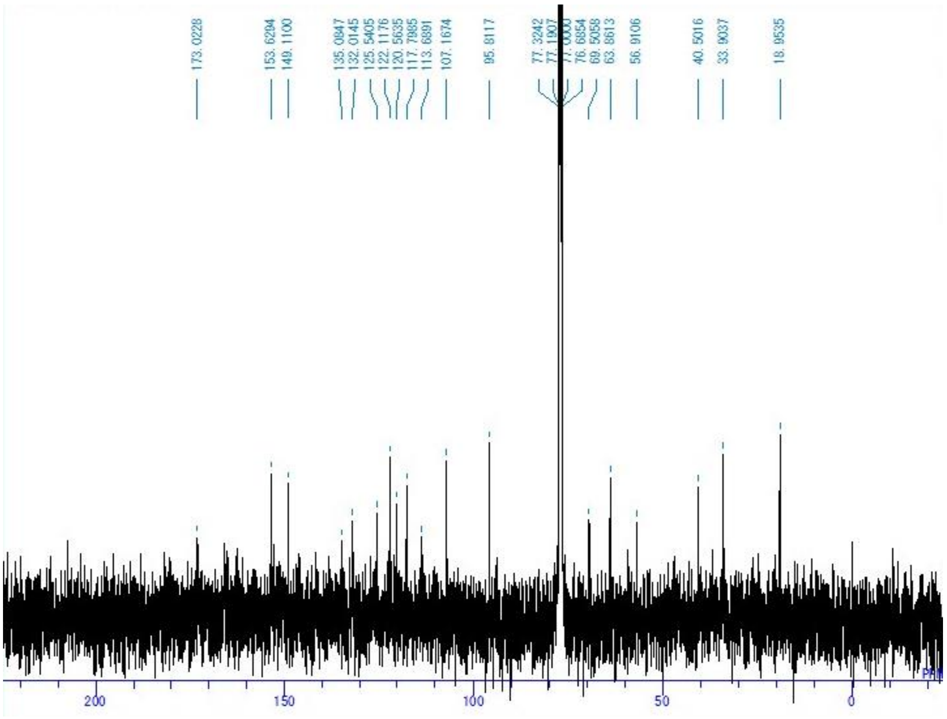

DDHK (**3**).

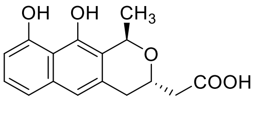

OH
OH
CH_3
O
COOH

3

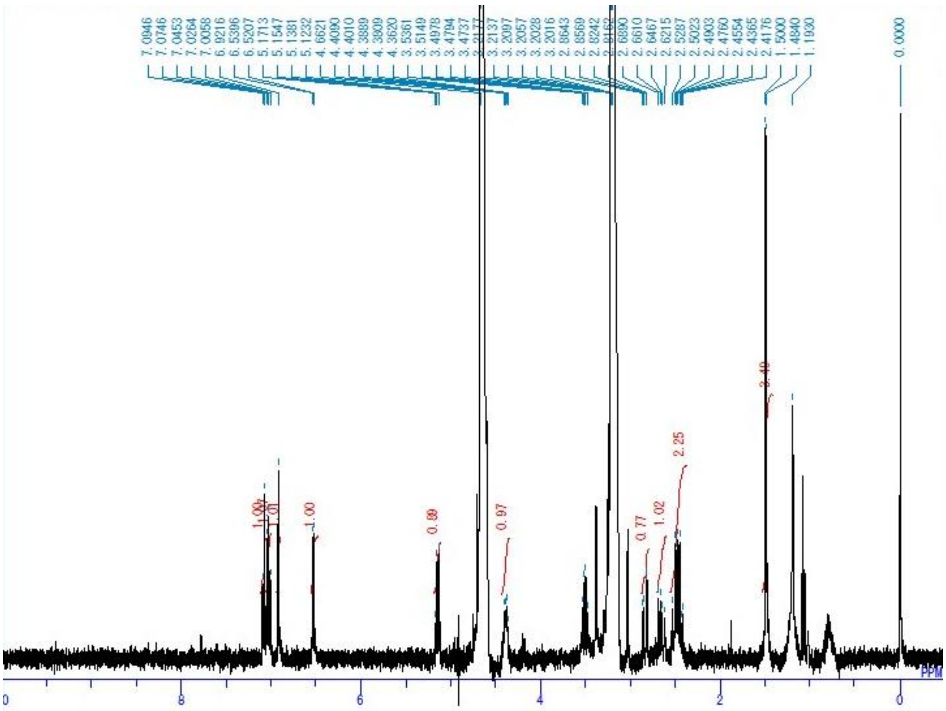

7.0946
7.0746
7.0453
7.0264
7.0058
6.9216
6.5396
6.5207
5.1713
5.1547
5.1381
5.1232
4.6621
4.4090
4.4010
4.3889
4.3809
4.3620
3.5361
3.5149
3.4978
3.4794
3.4737
3.2137
3.2097
3.2057
3.2028
3.2016
2.8643
2.8569
2.8242
2.6890
2.6610
2.6467
2.6215
2.5287
2.5023
2.4903
2.4760
2.4554
2.4365
2.4176
1.5000
1.4840
1.1930
0.0000
1.00
1.01
1.00
0.89
0.97
0.77
1.02
2.25
3.40
8
6
4
2
0
PPM

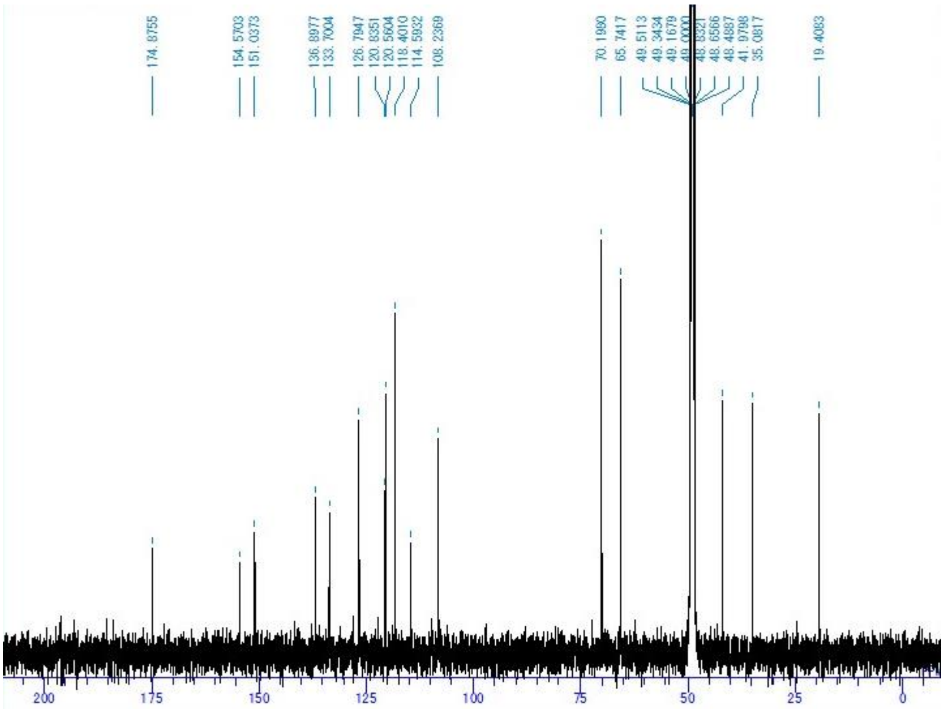

174.8755
154.5703
151.0373
136.8977
133.7004
126.7947
120.8351
120.5604
118.4010
114.5932
108.2369
70.1980
65.7417
49.5113
49.3434
49.1679
49.0000
48.8321
48.6566
48.4887
41.9798
35.0817
19.4083
200
175
150
125
100
75
50
25
0
